# Suicide and Head and Neck Cancer: A Systematic Review With Meta‐Analysis and Narrative Synthesis

**DOI:** 10.1002/pon.70233

**Published:** 2025-07-25

**Authors:** Jeffrey R. Hanna, Kairen McCloy, Jane Anderson, Angela McKeever, Cherith J. Semple

**Affiliations:** ^1^ Institute of Nursing and Health Research Ulster University Belfast UK; ^2^ Ulster Hospital South Eastern Health and Social Care Trust Dundonald UK; ^3^ Antrim Area Hospital Northern Health and Social Care Trust Antrim UK; ^4^ Department of Nursing University of Huddersfield Huddersfield UK; ^5^ School of Dentistry University of Leeds Leeds UK; ^6^ Altnagelvin Area Hospital Western Health and Social Care Trust Londonderry UK

**Keywords:** head and neck cancer, oncology, suicide, suicide completion, suicide ideation

## Abstract

**Objective:**

People with head and neck cancer are up to three times more likely to die by suicide than the general population. There is an urgency to understand and address the growing rates of suicidality within this population. The objectives of this review are (1) to explore the risk factors for thoughts of suicide and self‐harm, and suicide completion in patients with head and neck cancer, and (2) to understand the challenges and needs of patients impacted by head and neck cancer who have had thoughts of self‐harm and suicide.

**Methods:**

Mixed‐methods systematic review following the PRISMA protocol. Electronic databases and grey literature searches were completed using MeSH terms and key word searches. A total of 3665 recorded were identified; with 36 studies included. Of these, 22 focussed on suicide completion, with sufficient data to conduct a meta‐analysis on several important risk factors for suicide completion. These are sex, age, time since diagnosis and marital status. The remaining 14 studies reported on suicide ideation for this population, with the findings analysed within a narrative synthesis. Findings and clinical implications were refined with input from nine members of a head and neck cancer patient and public involvement group.

**Findings:**

Risk of suicide ideation and suicide completion was greatest in male patients. Suicide completion was highest in patients within the first 6‐months of diagnosis, who were widowed, or had cancer of the hypopharynx. Suboptimal pain and symptom management appeared related to a higher risk of suicide ideation. A therapeutic and supportive relationship with health and social care professionals was helpful in managing experiences of suicidal ideation.

**Conclusions:**

Health and social care professionals should identify, assess, support and follow‐up regarding thoughts of suicide for patients with head and neck cancer. Clear pathways are necessary for the management of suicidality, to include appropriate referrals to psychiatry/psychology, supportive interventions to include medications that can help with pain, distress or other symptoms.

## 1 Background

While global rates of suicide have been steadily decreasing [1], the rate of suicide is significantly higher among people impacted by cancer [2]. Consistently, studies have highlighted people living with and beyond head and neck cancer are at greater risk of suicidal ideation and death by suicide than the general population [3, 4, 5]. Suicidal ideation is operationally defined as thoughts of self‐harm and suicide [6]. Globally, the incidence of suicide among patients with head and neck cancer is up to three times that of the general population [7, 8, 9]. While survival rates in head and neck cancer are improving [10], statistics highlight that the rate of suicide in people living with and beyond head and neck cancer is increasing [1, 10, 11].

Treatments for head and neck cancer are complex, whereby individuals often undergo multi‐modality therapy, combining surgery, chemotherapy, and radiotherapy, resulting in challenging symptoms and persisting adverse treatment effects [12]. These include facial disfigurement, chronic functional impairments related to eating, swallowing, and speaking, and long‐term psychosocial issues such as anxiety, depression, and fear of recurrence which impact on overall quality of life [13, 14, 15]. As a result, adverse symptoms, coupled with functional and aesthetic issues have been shown to positively contribute towards the potential for suicidal ideation and death by suicide for this population [8].

The World Health Organization considers suicide prevention as a major public health priority, with Sustainable Development Goals of working towards reducing the rate of suicide by one‐third by 2030 [16]. Evidence‐based interventions can provide timely support and mitigate against the harmful effects of suicidal ideation [17]. Given the incidence of higher rates of suicidal ideation and death by suicide in patients with head and neck cancer has been widely reported [4, 7, 8, 9], this suggest intervention is required [18]. There is a need to understand those individuals who are at greater risk of suicidal ideation in head and neck cancer, and those who complete suicide to understand the populations most at risk and where intervention is of greatest need.

Therefore, the aim of this systematic review was to gain an enhanced understanding of risk factors, needs and experiences surrounding suicidality for people with head and neck cancer. Such knowledge can inform efforts on how best to support this population to reduce suicidal ideation and suicide completion.

### 1.1 Objectives

The objectives of the study are:

1. to determine the risk factors of suicidal ideation and suicide completion in people impacted by head and neck cancer,
2. to explore the challenges of people impacted by head and neck cancer who have had thoughts of suicide and self‐harm,
3. to explore the needs of people impacted by head and neck cancer who have had thoughts of self‐harm and suicide.

## 2 Methods

This systematic review followed an a priori protocol according to the New Preferred Reporting Items for Systematic Reviews and Meta‐Analyses Protocol (PRIMSA 2020) guidelines [19], which is an internationally recognised standardised guide that facilitated the development and reporting of this systematic review. The review protocol was registered on PROSPERO before the search was conducted (registration no. CRD42024521993).

### 2.1 Search Strategy

Existing literature was systematically searched to identify articles related to suicidal ideation and suicide completion in patients impacted by head and neck cancer. Four electronic databases (Ovid MEDLINE, Scopus, CINAHL and PsycINFO) were searched on April, week 4, 2024, using Medical Subject Heading (MeSH) terms and text word searches, to increase the search sensitivity. Boolean operators ‘OR’ and ‘AND’ were used to combine search terms to broaden or limit the search results, as appropriate. The search terms were generated in consultation with an experienced subject librarian and the first two authors [JRH + KMcC] using elements of the PICO framework [20]. The fully devised search strategy was independently peer‐reviewed using the PRESS tool [21] by two co‐authors [CJS + JA] (see appendix 1). The search strategy (Table 1) was deployed by the first author [JRH]. An updated search was then completed on February, week 2, 2025 identifying no new studies.

**TABLE 1:** Search strategy deployed within the four electronic databases.

PICOs category	Search strategy			
	Ovid medline	PsycINFO	CINAHL	Scopus
Population	1. exp caregivers/or exp patients/	1. exp caregivers/or exp patients/	1. Caregivers/or Patients/	
	2. Carer*.mp, or patient*.mp, or family care*.mp, or informal care*.mp, or spousal care*.mp	2. Carer*.mp, or patient*.mp, or family care*.mp, or informal care*.mp, or spousal care*.mp	2. ‘Carer*’, or ‘patient*’, or ‘family care*’, or ‘informal care*’, or ‘spousal care*’,	1. Carer* OR patient* OR ‘family care*’ OR ‘informal care*’OR “spousal care*
Context	3. exp Head and neck neoplasms/, or exp mouth neoplasms/ exp laryngeal neoplasms/ exp pharyngeal neoplasms/ exp oropharyngeal neoplasms/	3. exp neoplasms/	3. Head and neck neoplasms/, or pharyngeal neoplasms/, or oropharyngeal neoplasms/, or laryngeal neoplasms/, or mouth neoplasms/	
	4. head and neck cancer*.mp, or head and neck neoplasm*.mp, or head and neck tumo?r*.mp, or oral cancer*.mp, or oral neoplasm*.mp, or mouth cancer*.mp, or mouth neoplasm*.mp, or laryn* cancer*.mp, or laryn* neoplasm*.mp, or laryn* tumo?r*.mp, or pharyn* cancer*.mp, or pharyn* neoplasm*.mp, or pharyn* tumo?r*.mp, or carcinoma*.mp, or malignan*.mp, or throat cancer*.mp, or Oropharyn*.mp	4. head and neck cancer*.mp, or head and neck neoplasm*.mp, orhead and neck tumo?r*.mp, or oral cancer*.mp, or oral neoplasm*.mp, or mouth cancer*.mp, or mouth neoplasm*.mp, or laryn* cancer*.mp, or laryn* neoplasm*.mp, or laryn* tumo?r*.mp, or pharyn* cancer*.mp, or pharyn* neoplasm*.mp, or pharyn* tumo?r*.mp, or carcinoma*.mp, or malignan*.mp, or throat cancer*.mp, or Oropharyn*.mp	4. ‘head and neck cancer*’, or ‘head and neck neoplasm’, or ‘head and neck tumo?r*’, or ‘oral cancer*’, or ‘oral neoplasm*’, or ‘mouth cancer*’, or ‘mouth neoplasm*’, or ‘laryn* cancer*’, or ‘laryn* neoplasm*’, or ‘laryn* tumo?r*’, or ‘pharyn* cancer*’, or ‘pharyn* neoplasm*’, or ‘pharyn* tumo?r*’, or ‘carcinoma*’,or ‘malignan*’, or ‘throat cancer*”, or “Oropharyn*:	2. ‘head and neck cancer*’ OR ‘head and neck neoplasm*’ OR ‘head and neck tumo?r*’ OR ‘oral cancer*’ OR ‘oral neoplasm*’ OR ‘mouth cancer*’ OR ‘mouth neoplasm*’ OR ‘laryn* cancer*’ OR ‘laryn* neoplasm*’ OR ‘laryn* tumo?r*’ OR ‘pharyn* cancer*’ OR ‘pharyn* neoplasm*’ OR ‘pharyn* tumo?r*’ OR carcinoma* OR malignan* OR ‘throat cancer*’ OR Oropharyn* OR Otorhinolargn*
Outcome	5. exp suicidal ideation/ exp suicide, Attempted/ exp suicide, completed/ exp. Suicide prevention/ exp suicide/exp self‐Injurious behaviour/	5. exp suicidal ideation/ exp Attempted suicide/ exp. Suicide prevention/ exp suicide/	5. Suicidal ideation/ suicide, attempted/ suicide prevention/ suicide/ self‐injurious behaviour	
	6. suicid*.mp, or self harm.mp, or suicid* ideation.mp, or self injur*.mp, or self‐destruct*.mp, or self‐poison*.mp	6. suicid*.mp, or self harm.mp, or suicid* ideation.mp, or self injur*.mp, or self‐destruct*.mp, or self‐poison*.mp	6. ‘suicid*’, or ‘self?harm’, or ‘suicide* ideation’, or ‘self injur*’, or ‘self‐destruct*’, or ‘self‐poison*’	3. suicide* OR ‘self harm*’ OR ‘suicid* ideation’ OR ‘self injur*’ OR ‘ self destruct*’ OR ‘ self poison*’
	7. 1 OR 2 AND 3 OR 4 AND 5 OR 6	7. 1 OR 2 AND 3 OR 4 AND 5 OR 6	7. 1 OR 2 AND 3 OR 4 AND 5 OR 6	4. 1 AND 2 AND 3
	8. Limit 7 to English	8. Limit 7 to English	8. Limit 7 to English	5. Limit 4 to English

Grey literature searches were conducted using the term ‘head and neck cancer’ and ‘suicide’ on Google Scholar (first 100 results) and TRIP Medical Database, as well as handsearching of relevant journals, to identify research not indexed in the electronic databases.

### 2.2 Study Inclusion and Exclusion Criteria

A list of inclusion and exclusion criteria was applied to ensure only studies relevant to the aims and objectives of the review were included (Table 2). This included qualitative, quantitative, or mixed‐method studies using any research design and analysis that explored suicidal ideation or suicide completion in patients, who were aged 18 years or older, and impacted by head and neck cancer. Only studies published in the English language were included. No restrictions were applied to the location of research or year of publication to have a comprehensive understanding of the global literature.

**TABLE 2:** Study inclusion and exclusion criteria.

	Inclusion	Exclusion
Study design	Quantitative, qualitative, or mixed‐method studies using any research design.	Opinion pieces, systematic reviews, commentaries, editorials.
Participants	Patients who have, or had, a diagnosis of head and neck cancer. OR Carers (spouse/partner, family member, friend or neighbour) of individuals who have head and neck cancer. Individuals ≥ 18 years old. Studies that report on risk factors, challenges, or needs for suicidal ideation. Studies that report on risk factors for suicide completion.	Individuals < 18 years old. Individuals with a cancer diagnosis other than head and neck cancer.

### 2.3 Screening

**FIGURE 1:**
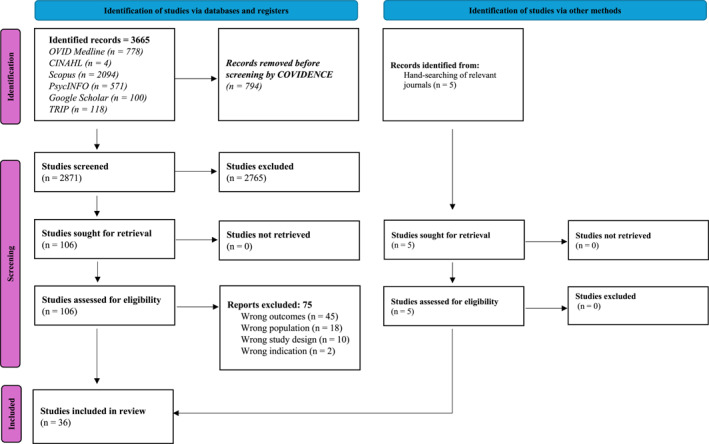
Preferred reporting items for systematic reviews and meta‐analysis (PRISMA)[19].

The electronic searches identified 3665 publications which were uploaded to COVIDENCE to manage the screening process, of which 794 duplicates were automatically removed. The remaining 2871 publications were reviewed by title and abstract by the first author [JRH]. To promote rigour and transparency, publications were independently reviewed at title and abstract level by either JA (51%) or KMcC (49%). There were no conflicts of interest in this process, and a total of 2765 publications were identified as irrelevant. Full‐text publications were then uploaded to COVIDENCE for the remaining 106 publications deemed eligible based on title and abstract. Publications were reviewed at full‐text stage by the first author [JRH], with independent second member screening by either KMcC (56%) or JA (44%). There were no conflicts in the process of screening at full‐text stage, with 75 publications being excluded. An additional five records were subsequently identified through handsearching of relevant journals by CJS and JRH. A total of 36 studies were eligible for inclusion in the review. The PRISMA‐P diagram illustrates this process with documented explanations for excluded publications at the full‐text stage (Figure 1).

### 2.4 Data Extraction and Quality Assessment of Included Studies

Data were independently extracted using an extraction sheet in Microsoft Excel (2024) by JRH. Data were extracted on the author(s), year, country, study aims and objectives, sample characteristics, and key findings relevant to the review's aims and objectives. To promote rigour and transparency, extracted data was independently verified by the second author [KMcC]. There were no conflicts regarding the extracted data.

A critical evaluation was conducted to assess the appropriateness, acceptability, biases, and validity and reliability of the design, conduct and analysis within the 36 studies. A variety of approaches for evaluation were used to assess the studies that were appropriate to the study design. This included the Critical Appraisal Skills Programme (CASP) quality assessment tool for cohort studies (*n* = 29), the Joanna Briggs Institute (JBI) Critical Appraisal Checklist for Case Reports (*n* = 3), the JBI Checklist for Quasi‐Experimental Studies (*n* = 1), and the JBI Critical Appraisal Checklist for Analytical Cross‐Sectional Studies (*n* = 3). This process was completed independently by AMcK and JRH, with CJS consulted to resolve disagreements. See appendix 2 for completed quality assessments.

### 2.5 Data Analysis

#### 2.5.1 Suicide Completion

Following extraction from the 36 studies, 22 studies focussed on suicide completion in head and neck cancer. There was sufficient data to conduct a meta‐analysis on several important risk factors for suicide completion in head and neck cancer. This included sex, age, time since diagnosis, and marital status. Standardised mortality ratios and hazard ratios were treated as equivalent measures of risk [22, 23]. Sex was categorised as male and female as defined within the studies. As frequently dichotomised in the studies, age was categorised into two groups: up to 54 years and over 55–79 years. Four categories were identified for time since diagnosis: 0–5 months, 6 months to 1 year, 1–2 years and 3–10 years. Marital status included four categories: married, single‐never married, divorced and widowed. Revman Web was used to conduct the meta‐analysis, using inverse‐variance weighted DerSimonian–Laird random‐effect models to allow for between‐study heterogeneity [24]. Between‐study heterogeneity was quantified using Cochran's Q [25]and the *I* ^2^ statistic [26]. *I* ^2^ can be interpreted as the proportion of the total variation in the estimated effects for each study that is due to heterogeneity between studies [26]. Heterogeneity was evaluated using the *I* ^2^ statistics with values of 0%–40% representing ‘might not be important’, 30%–60% representing ‘moderate’, 50%–90% ‘substantial’, and 75%–100% ‘considerable’ heterogeneity [27]. If substantial or considerable heterogeneity was evident while conducting meta‐analysis, then sensitivity analysis was also performed to explore reasoning.

Examination of the broad extraction sheet identified three risk factors for suicide completion in head and neck cancer (psychiatric symptomatology, HPV status and cancer staging) that were not suitable for a meta‐analytic approach because there was insufficient data to include. Alongside this, it was not possible to conduct a meta‐analysis on tumour site as a risk factor for suicide completion due to inconsistent reporting within the studies. Where appropriate, the second author [KMcC] contacted the first authors of studies to obtain additional or unpublished data but received no response.

#### 2.5.2 Suicidal Ideation

Due to insufficient, inconsistent and infrequent reporting of data for suicidal ideation in head and neck cancer across 14 studies, it was deemed appropriate to conduct a narrative synthesis on this data. The narrative synthesis was conducted in accordance with Poppay and colleagues [28] guidance on conducting a narrative synthesis in systematic reviews and the principles of Thomas and Harden's thematic synthesis [29]. Initially, the first author [JRH] coded the extracted qualitative data by using similar words and phrases within the text. Quantitative data was converted to qualitative codes that reflected the meaning of the data. This process was managed using Microsoft Excel (2024). Codes were independently reviewed by the second author [KMcC]. Then, JRH identified were some of the codes grouped together as themes that told the *‘story’* of the data. The themes were independently reviewed by the last author [CJS] and refined through critical dialogue with the first author [JRH]. All authors reviewed and agreed to the final themes and were ‘sense checked’ with a head and neck cancer patient and public involvement group on two occasions.

The themes were then assessed using the GRADE—Confidence in Evidence from Reviews of Qualitative Research (GRADE‐CERQual) to assess the methodological limitations, coherence, relevance and adequacy of the review findings [30]. The level of confidence reflects the extent to which the review findings represent the topic of interest and its application in the development of guidelines and policy [30]. The GRADE—CERQual was applied by the first author [JRH] and confirmed by the research team.

### 2.6 Quality Assessment of the Evidence

To determine the studies as having ‘high’, ‘moderate’ or ‘low’ quality, the GRADE approach to assessing evidence quality was applied to each study [31]. Aligned to established thresholds that indicate quality assessment, it was agreed as a research team that the presence of > 80% of the appraisal criteria within each assessment would be considered as ‘high’ quality, 50%–79% to be ‘moderate’ quality, and < 49 as ‘low’ quality [31, 32]. Overall, 25 of the studies were rated as ‘high’ quality, with six rated as ‘moderate’ quality (see appendix 2). Furthermore, all themes within the narrative synthesis received a ‘moderate’ confidence rating following application of the GRADE—CERQual [30] (see appendix 3). No studies were rated of low quality; hence no studies were excluded from data synthesis due to poor quality.

### 2.7 Patient and Public Involvement

An existing head and neck cancer patient and public involvement group comprising of 21 patients and carers living with and beyond head and neck cancer were consulted on two occasions. First, six members of the group provided feedforward to the review findings during an in‐person event in June 2024, where the first author [JRH] provided the group with a presentation of the findings from the 36 included studies. Second, the group were invited to provide written feedback to the clinical implications in November 2024. A total of 9 members of the group responded to the invitation and helped to shape the clinical recommendations section, by offering additional suggestions that were not considered by the research team.

## 3 Results

### 3.1 Overview of the Studies

The 36 studies were conducted between 2003 and 2024 across 13 countries. Of the 36 studies, 22 (61%) were published within the last 5‐years [4, 9, 18, 33, 34, 35, 36, 37, 38, 39, 40, 41, 42, 43, 44, 45, 46, 47, 48, 49, 50, 51]. Collectively, the studies report on 50,358,040 cancer cases. While the studies reported hazard ratios or standardized mortality rates relevant to the objectives of this review, nine of the studies did not detail the total number of patients with a head and neck cancer diagnosis [43, 44, 51, 52, 53, 54, 55, 56, 57]. Excluding these nine studies, there were 28,975,396 cancer patients reported across 27‐studies, of which 2,108,483 (7.2%) had a head and neck cancer diagnosis. Fourteen of the studies are reflective of suicidal ideation in patients with head and neck cancer [35, 36, 37, 38, 40, 45, 46, 47, 48, 50, 58, 59, 60, 61]. The other 22 studies are reflective of risk factors for suicide completion in patients with head and neck cancer [4, 7, 8, 9, 18, 33, 34, 39, 41, 42, 43, 44, 45, 49, 51, 52, 53, 54, 55, 56, 57, 62, 63]. Characteristics for each of the included studies including factors such as gender, age, diagnosis and tumour sites are presented within Table 3.

**TABLE 3:** Characteristics of included studies.

Author(s), year and country	Study aim(s)	Research design	Suicide‐related outcome	Sample and sociodemographic details relevant to this review	Key findings relevant to aims and objectives	Quality assessment
Akechi et al. [58], 2010 and Japan	To investigate gender differences in the backgrounds of cancer patients (age, marital status, employment status, cancer site, disease stage, performance status, brain metastasis, inpatient status, pain, severity of depression, and psychotic feature) suffering from major depression with or without suicidal ideation.	Cohort study	Suicide ideation	68 people with a head and neck cancer diagnosis also diagnosed with major depression.	Suicidal ideation was highest in males (45/68) with head and neck cancer compared to females.	High
Chakoma et al. [33], 2023 and USA	To investigate the association between HPV tumour status and suicide risk among patients with head and neck cancer.	Cohort study	Suicide completion	Database one: 60,361 patients diagnosed with head and neck cancer between 2010 and 2017. Of the 160 suicides recorded in this cohort, 69 were among individuals with HPV‐positive head and neck cancer and 91 among those with HPV‐negative head and neck cancer. Database two: 125,477 patients diagnosed with head and neck cancer between 2000 and 2018. A total of 180 deaths by suicide were among patients with HPV‐related cancer and 226 deaths by suicide among patients with non–HPV‐related cancers.	There was no association between HPV‐associated tumour sites and suicide. Positive HPV status was associated with increased suicide risk. Suicide rates in HPV‐related and non–HPV‐related head and neck cancer were higher than those of the general population. Factors associated with suicide risk in head and neck cancer included being male, unmarried, lack of surgical treatment, and lack of radiotherapy.	High
Chang et al. [35], 2019 and Taiwan	To investigate the risk factors of suicidal ideation among head and neck cancer patients, giving more evidence for arranging intervention in the future.	Longitudinal study	Suicide ideation	286 patients with head and neck cancer who were referred for psychiatric consultation due to poor physical and psychosocial status. Of these, 27 were diagnosed as having suicidal ideation.	Suicidal ideation was greater in males (26/27) compared to females. The most frequent cancer site was the oral cavity (15/27), followed by the hypopharyngeal (5/27) and the oropharyngeal (4/27). In the suicidal ideation group, over half of patients were diagnosed with depression (14/27). Patients with head and neck cancer who experienced dysphoria had an eight times higher risk of suicidal ideation than those without these symptoms. Patients with hypopharyngeal cancer had a 13‐fold increased risk of suicide. Patients with a previous cancer history were at increased risk of suicidal ideation.	High
Chang et al. [36], 2022 and Taiwan	To provide insights into possible links between demoralization among oral cancer patients and its effects on the patient's spiritual needs, quality of life, and suicidal ideation.	Cross‐sectional study	Suicide ideation	155 inpatients with oral cancer: 147 males and 8 females. Tumour sites included tongue (*n* = 61), buccal (*n* = 44), gum (*n* = 26), mouth (*n* = 19) lip (*n* = 4), oropharynx (*n* = 1).	The odds ratio of suicidal ideation was 20x more for patients with higher demoralization than for those with lower demoralization. Cancer status, treatment duration, and recurrence status were not significantly associated with demoralization.	High
Choi and park [9], 2021 and South Korea	To determine whether suicide risk increases after a cancer diagnosis.	Cohort study	Suicide completion	A total of 2054 head and neck cancer patients (lip, oral cavity and pharynx), of which 11 died by suicide (9 males/2 females).	The relative risk of suicide was increased substantially for male patients with lip, oral cavity and pharyngeal cancer compared with the cancer free group.	High
Choi et al. [34], 2022 and South Korea	To examine the association between mental disorders and suicide risk among cancer patients.	Cohort study	Suicide completion	7706 patients with head and neck cancer, of which 5162 did not have a mental disorder, and 11 died by suicide. 2544 patients with head and neck cancer had a mental disorder, and 9 died by suicide.	There was no increased risk of suicide observed in head and neck cancer patients across those with or without a mental disorder.	High
Goelitz et al. [59], 2003 and USA	To report on the case of a terminally ill patient who expresses suicidal ideation.	Case study	Suicide ideation	49‐year‐old patient with head and neck cancer.	Suicidal ideation weas identified following referral to psychiatry for capacity assessment. Suicidal ideation appeared connected to uncontrolled pain and symptom management. This included dysarthria. Immediate care needs included obtaining financial resources and adequate pain relief. Follow‐up appointments were occasionally missed that appeared related to uncontrolled pain and symptoms. Referrals to psychology were not necessary as the suicidal ideation was connected to inadequate pain and symptom management. Lack of available support networks to provide practical and psychological help. Important for health and social care professionals to follow‐up when appointments are missed to promote a sense of being care for by healthcare system. Bi‐weekly follow‐up appointments were considered appropriate in situations of suicidal ideation.	Moderate
Hem et al. [52], 2004 and Norway	To determine whether cancer patients had a higher suicide risk between 1960 and 1999	Cohort study	Suicide completion	21 males and 6 females with cancer of the buccal cavity and pharynx died by suicide.	Suicide was 3x greater in males with cancer of the buccal cavity compared to females	High
Henry et al. [60], 2018 and Canada	(1) To determine the 1‐year period prevalence of suicidal‐ideation, suicide attempt, and completed suicide among patients newly diagnosed with a first occurrence of head and neck cancer. (2) to characterise stability and trajectory of suicidal ideation over the year following cancer diagnosis. (3) identify patients at risk of suicidal ideation.	Longitudinal study	Suicide ideation	223 patients with head and neck cancer who were newly diagnosed (< 2 weeks) with a first occurrence of primary head and neck cancer were included.	Patients with head and neck cancer who were most susceptible to being suicidal in the first‐year post‐diagnosis presented with a self‐reported psychiatric history, with a 2x risk of suicidal ideation and were coping with their cancer diagnosis with substance use. Patients were equally likely to be suicidal at all time points. 49% of suicidal patients presented a score of > 3 on the beck scale for suicidal ideation upon being diagnosed with head and neck cancer, compared with 69.2% at 3‐month, 50% at 6 months and 46.2% at 12‐month.	High
Henry et al. [38], 2022 and Canada	(1) To characterise the sociodemographic, psychological, and social profiles of patients with HPV‐positive versus negative squamous cell carcinoma of the head and neck. (2) to identify how HPV status contributes to anxiety and depression, quality of life and sexuality needs.	Longitudinal study	Suicide ideation	79 patients with HPV‐positive cancers and 67 patients with HPV‐negative cancers. Tumour sites included: Nasopharyngeal (*n* = 82), oral (*n* = 46), oropharyngeal (*n* = 11), and hypopharyngeal (*n* = 7). 99/146 of the sample were male.	Patients with HPV‐negative cancers were more likely to have lifetime pre‐cancer suicidal ideation (11/16) than patients who were HPV‐positive (5/16). Patients with a lifetime history of suicidal ideation presented immediately post‐treatment with lower levels of quality of life.	High
Henry et al. [37], 2022 and Canada	In patients newly diagnosed with head and neck cancer: (1) the prevalence, level and course of body image concerns; (2) correlates of upon cancer diagnosis (pre‐treatment) body image concerns; (3) predictors of immediate post‐treatment body image concerns; and (4) association between body image concerns and levels of anxiety, depression, suicidal ideation, support, and alcohol and drug misuse.	Longitudinal study	Suicide ideation	219 patients with head and neck cancer. 151 were male and the mean age was 63 years. Cancer sites included: Oropharynx (*n* = 80), oral (*n* = 44), larynx (*n* = 37), skin (*n* = 15), nasopharynx (*n* = 18), unknown primary (*n* = 12) and other (salivary glands, paranasal sinuses, and nasal cavity (*n* = 13). 149/219 participants completed the 3‐month follow‐up.	Correlates of body image concerns in patients with head and neck cancer at baseline were significantly associated with suicidal ideation prior to diagnosis. Body image concerns in the immediate post‐treatment were significantly associated with suicidal ideation.	High
Henson et al. [39], 2019 and UK	To quantify suicide risk in patients with cancer in England and identify risk factors that may assist in needs‐based psychological assessment.	Cohort study	Suicide completion	167,307 patients with a head and neck cancer diagnosis, of which 176 died by suicide.	The absolute excess risk for males with head and neck cancer was significantly greater than for females.	High
Heyda et al. [40], 2023 and Poland	To show the multilevel and complex cooperation and the inclusion of the psychotherapist leading the psychotherapy in the medical team at a radiotherapy and clinical oncology clinic.	Case study	Suicide ideation	A 43‐year‐old male diagnosed with advanced head and neck cancer during the COVID‐19 pandemic. The individual had pre‐existing mental health problems, including OCD, PTSD and psychoactive substance abuse.	The patient directly expressed suicidal ideation to the medical doctor. The patient had a complex mental health history. It was considered necessary for this patient to have appropriate medication prescribed until an appointment with psychological services could be facilitated. It was important for health and social care professionals to routinely follow‐up and re‐assess for suicidal thoughts. Psychotherapy was facilitated which involved daily 45‐min sessions with a psychotherapist during a 7‐week period of radiotherapy. Symptoms of PTSD were reported as reduced following the intervention.	High
Hu et al. [41], 2023 and USA	To provide contemporary estimates of suicide risks associated with cancer and to identify sociodemographic and clinical factors associated with suicide risks among individuals diagnosed with cancer.	Cohort study	Suicide completion	409,033 patients diagnosed with oral cavity or pharynx cancer. Of these, 1157 died by suicide.	Higher suicide risks were seen for patients with oral cavity and pharynx during the first 2 years of diagnosis.	High
Innos et al. [53], 2003 and Estonia	To determine the suicide risk among cancer patients in Estonia.	Cohort study	Suicide completion	17 patients with a head and neck cancer diagnosis who died by suicide.	A significant increased suicide risk was seen in male patients with cancer of the lip, oral cavity and pharynx (16/17) compared to females (1/17). Risk of suicide was highest between 90 and 179 days of follow‐up for both males and females with head and neck cancer.	High
Kam et al. [8], 2015 and USA	To identify incidence rate, trends, and risk factors of suicide in patients with cancer of the head and neck.	Cohort study	Suicide completion	350,413 patients diagnosed with head and neck cancer between 1973 and 2011. Of these, 857 died by suicide.	Suicide rates were statistically significantly higher in male patients and those with later stage disease. Those diagnosed at ages 60–79 years had the highest rate of suicide. Patients who received only radiation had approximately double the suicide rate, than those who received only surgery. Hypopharyngeal cancers were associated with the highest suicide rates in both men and women. The greatest increase in suicide rates among all patients with head and neck cancer was seen in the first 5 years after diagnosis, with a subsequent decline over time.	High
Kapoor and bhatnagar [61], 2018 and India	A case study of a patient being treated for oral cancer who suffered from severe mental distress and made a suicide attempt.	Case study	Suicide ideation	55‐year‐old male with mouth cancer.	The patient reported a suicide attempt to the medical professional. The suicide attempt appeared related to uncontrolled pain and symptom management. This included burning sensations as a consequence of radiotherapy. It was appropriate to ensure adequate pain management and provision of education on how to manage symptoms. Follow‐up appointments were occasionally missed which was attributed to uncontrolled pain and symptom management. A referral to psychology was considered unnecessary, with a focus on optimising pain relief and adequate symptom management. A supportive family network was noted. Daily counselling was offered while treated as inpatient. Suicidal ideation was reported as reduced once pain and symptoms were controlled.	Moderate
Kendal [62], 2007 and Canada	A population‐based analysis of cancer patients, comparing suicide risk between the genders to elucidate the features specific to each gender.	Cohort study	Suicide completion	13,756 females diagnosed with head and neck cancer, of which 7 died by suicide. 30,811 males were diagnosed with head and neck cancer, of which 99 died by suicide.	Males with head and neck cancer had increased rates of suicide. White individuals with head and neck cancers exhibited a greater suicide hazard. The suicide risk with these head and neck cancer was also increased with age and was decreased with married status.	High
Liu et al. [43], 2022 and USA	To investigate suicide risk among patients with different cancer types in the United States and to identify subsets of patients at particularly high risk.	Cohort study	Suicide completion	14,423 cancer patients were identified as having died by suicide.	The greatest risk of suicide occurred in patients with cancer of the hypopharynx in the first 2 months following diagnosis. In cancers of the oral cavity and pharynx, males demonstrated a higher standardised mortality ratio and absolute excess risk than women. Patients who identified as black demonstrated the highest standardised mortality rate in cancer of the oral cavity and pharynx.	High
Liu et al. [42], 2023 and Taiwan	To understand the suicide risk of patients with head and neck cancer in Taiwan compared with patients with other‐cancer and general population during the period from 2010 to 2019.	Cohort study	Suicide completion	74,495 patients newly diagnosed with head and neck cancer between 2011 and 2017. A total of 396 died by suicide.	Males had a higher risk of suicide compared to females. Patients less than 56‐year were at greater risk of suicide compared to patients greater than 56‐year. Patients who are middle‐aged (50–60 years) have the highest suicide mortality risk. However, patients aged < 40 years have the lowest suicide mortality risk. Patients with the highest income group > 30,000 $NTD had a greater risk of suicide compared to those who earner between 15,000 and 30,000 $NTD and those who earned < 30,000 $NTD.	High
Michalek et al. [44], 2023 and Poland	To explore whether Polish cancer patients face elevated suicide risk.	Cohort study	Suicide completion	830 people who died by suicide; 49 had a head and neck cancer diagnosis.	The risk of death by suicide was significantly higher in males with head and neck cancer. A higher risk of suicide risk was increased for females with tumours of the larynx. The risk of suicide was highest within the first 6 months after diagnosis for patients with head and neck cancer. Also, between six and 12 months there was an increased risk. From the fifth to the tenth‐year post‐diagnosis, there was an increased risk in death by suicide for patients with head and neck cancer.	High
Misono et al. [63], 2008 and USA	To characterise suicide rates among patients with cancer in the USA and identify patient and disease characteristics associated with higher suicide rates.	Cohort study	Suicide completion	76,331 people with an oral cavity, pharynx or larynx cancer diagnosis; 291 died by suicide	The rate of suicide in this population was greatest within the first‐five years of diagnosis.	High
Nasseri et al. [54], 2012 and USA	To measure suicide risk in cancer patients and compare it with the general population.	Cohort study	Suicide completion	1168 individuals died by suicide, which included 61 patients with cancer of the oral cavity.	Males with cancer of the oral cavity were more likely to die by suicide (59/61) than females (2/61). Risk of suicide in cancer of the oral cavity is greatest in White males (45/59) and White females (2/2).	High
Nugent et al. [45], 2021 and USA	To examine the associations between pre‐cancer mental health and pain and post‐cancer receipt of mental health, substance use disorders, or palliative care services with risk of suicidal self‐directed violence.	Cohort study	Suicide ideation	7803 survivors of head and neck cancer between 2012 and 2018, of which 51 died by suicide. The sample were predominately male (*n* = 7685) and identified as non‐Hispanic White (*n* = 6179). The most common primary tumour was pharyngeal (*n* = 3439) and laryngeal (*n* = 2365).	The most common means of suicidal self‐directed violence were intentional self‐poisoning with various substances (*n* = 28; 14 of whom died) or firearm discharge (*n* = 33, all of whom died). Almost half (*n* = 32) of suicidal self‐directed violence events were more than 2 years after the diagnosis; 18 (25%) had an event within 6‐month of their cancer diagnosis.	High
Oberaigner et al. [55], 2014 and Austria	To investigate whether suicide risk in Tyrol/Austria was increased for cancer patients as compared to the general population and whether subgroups at excess risk could be defined.	Cohort study	Suicide completion	53,803 cancer patients.	12 males and 1 female with head and neck cancer died by suicide.	High
Osazuwa‐Peters et al. [7], 2018 and USA	To determine whether gender and human papillomavirus‐relatedness are associated with increased risks of suicide in the head and neck cancer population.	Cohort study	Suicide completion	287,901 patients > 18 years diagnosed with head and neck cancer between 1973–2014, of which 1036 died by suicide.	92% of those who died by suicide were male and 90% were non‐Hispanic White. 60% of patients who died by suicide were 60‐year plus. Male head and neck cancer survivors were 6x more likely to die by suicide compared with females. HPV‐relatedness was not a significant predictor of suicide. Compared with non‐Hispanic White survivors, Hispanic, non‐Hispanic black and non‐Hispanic Other were less likely to die by suicide. Compared with married patients, there was a significantly increased risk of suicide among the widowed and those divorced/separated. There was an increasing risk of suicide with increasing age, from 40 to 59 years, 60–69 years, and 70+, compared with patients aged 18–39. Patients diagnosed with regional stage disease were 76% more likely to die by suicide compared to those with localised disease. Patients who received any single treatment or any combination of surgery, radiation and chemotherapy were less likely to die by suicide compared to patients who received no treatment. Patients with hypopharynx, nasopharynx, and oropharynx cancer were more likely compared to those with oral cavity cancer.	High
Osazuwa‐Peters et al. [4], 2021 and USA	To determine whether the risk of suicide among patients with head and neck cancer differs by rural versus. urban or metropolitan residence status.	Cross‐sectional study	Suicide completion	134,510 patients diagnosed with head and neck cancer between 2000 and 2016. Of these, 101,142 were male, the mean age was 57.7 years and 405 died by suicide.	Most of the suicides were non‐Hispanic White individuals (90.1%) and male (93.1%). The median time from diagnosis to death by suicide was 26 months (range, 0–187 months). The incidence of suicide was highest among rural patients with head and neck cancer.	Moderate
Smailyte et al. [56], 2013 and Lithuania	To estimate suicide risk and its sociodemographic determinants among cancer patients in Lithuania.	Cohort study	Suicide completion	215 patients with cancer who died by suicide.	A significantly increased risk of suicide was found among males with cancer of the buccal cavity and pharynx. There were no recorded deaths by suicide in females with cancer of the buccal cavity and pharynx. A total of 5 males and 0 females died by suicide who had cancer of the larynx.	High
Stanbouly et al. [46], 2023 and USA	To determine whether health insurance impacts the risk of suicidal ideation among patients with head and neck cancer	Cohort study	Suicide ideation	134,510 patients (> 18 years) of which 29,231 had a diagnosis of head and neck cancer, and 102 were identified as having suicidal ideation; most of whom were male (77/102), White (75/102) and > 60 years (51/102).	Age group, health insurance, oral cavity cancer, alcohol dependence/abuse, nicotine dependence, depressive episode, anxiety disorder and pain were all significant predictors of suicidal ideation. Relative to patients who were privately insured, patients with Medicaid were 2.44x more likely to experience suicidal ideation. Relative to other head and neck cancer sites, the oral cavity was associated with a decreased risk for suicidal ideation. Patients with alcohol dependent/abuse were at increased risk of suicidal ideation. Patients with depression were 8.30x more likely to experience suicidal ideation.	Moderate
Sun et al. [48], 2020 and Taiwan	To determine the risk of suicide attempts in patients with head and neck cancer in comparison with that in the general population and in patients of other cancers	Cohort study	Suicide ideation	Patients diagnosed with head and neck cancer between 2000 and 2010 compared with patients without head and neck cancer during the same period. A total of 66,931 patients were included in both groups.	Patients with oropharynx, hypopharynx and larynx cancer presented a higher risk of suicide attempt than individuals without head and neck cancer. The risk of suicide attempt among males was 1.60x higher than among females. Patients with depression exhibited a higher risk of suicide attempt than those without depression. Patients without depression, alcohol‐related illness, and other comorbidities (anxiety, insomnia, mental disorders, or schizophrenia), and male patients in the head and neck cancer group were associated with a significantly higher risk of suicide attempt than their counterparts. Chemotherapy was associated with a 1.58‐fold increase in the risk of suicide attempt among patients with head and neck cancer.	High
Sun et al. [47], 2023 and China	To investigate the current situation of economic toxicity and suicide risk in patients with head and neck cancer, analyse the relationship between them, and provide a new theoretical basis for the development of intervention programme to reduce the economic toxicity and suicide risk in patients with head and neck cancer.	Cross‐sectional study	Suicide ideation	150 patients with head and neck cancer. Of these, 102 were male; 48 were female. Diagnoses included: nasopharyngeal (*n* = 27), oral (*n* = 20), tongue (*n* = 31), submaxillary (*n* = 8), laryngeal (*n* = 7), hypopharyngeal (*n* = 9) and other related head and neck (*n* = 48) cancers.	Patients with economic toxicity (135/150) had significantly higher suicide risk scores than those without economic toxicity (15/150). Patients with hypopharyngeal carcinoma (9/150) had the highest total score of suicide risk. In terms of employment, the highest scores were reported among those who worked in enterprise (35/150). Other factors included not having commercial insurance (121/150), undergoing targeted therapy (83/150), less than 2 years since diagnosis (62/150), with a decrease between 2 and 5 years (52/150), and a slight increase again after 5‐year (36/150). Patients with less than < 3000 yuan of monthly income were at increased risk of suicide (44/150).	Moderate
Thavarajah et al. [18], 2019 and India	To describe survival (5‐year), mortality and suicide among patients with mid‐facial head and neck cancer and to identify the patient and disease characteristics associated with higher suicide rates.	Cohort study	Suicide completion	218,048 patients with head and neck cancer. Of these, 774 died by suicide.	Of the 774 head and neck cancer patients that died by suicided, 710 were male and 64 were female. Most of whom were married (426) with the most common tumour site being the tongue (191).	High
Tu et al. [49], 2023, China and USA	To develop an online risk stratification system, named ‘Larysuicide’, to identify patients at high risk of suicide after the laryngeal cancer diagnosis.	Cohort study	Suicide completion	Database one: 42,066 patients in the USA diagnosed with laryngeal cancer between 2000 and 2018. Of these, 594 patients died by suicide Database two: 4207 patients diagnosed with laryngeal cancer in China between 2010 and 2022. Of these, 55 patients died by suicide.	Of the USA population cohort that died by suicide, patients were predominately 60 years and older (451/594), white (444/594), with unknown stage (384/594), unknown grade (319/594), no surgery (387/594), and with radiation (466/594). There were no significant differences in relation to having chemotherapy, marital status or sex. In the Chinese cohort, patients who died by suicide were predominately 60 years and older (49/55), with unknown stage (39/55), and unknown grade. It was identified that the strongest predictors of death by suicide in patients with laryngeal cancer are individuals who are: > 60 years old, White, unknown grade, unknown stage and had radiotherapy.	High
Waltho et al. [50], 2021 and UK	To develop and evaluate a pathway for patients with head and neck cancer to screen for depression and suicidal ideation and deliver treatment for depression using a nurse led collaborative care intervention in order to meet the recommendations for psychological support.	Pilot intervention study	Suicide ideation	19 patients with head and neck cancer, of which six had suicidal ideation.	Pilot intervention study. Six sessions offered with an oncology nurse who had been extensively trained to the role. Key components of the intervention included psychiatric assessment, conversations with the patient about their emotional state, liaising with multi‐disciplinary team professionals for medication reviews and referral to psychosocial support services such as charity organisations. Scores for suicidal ideation were reported as reduced following partial or full completion of the six sessions.	Moderate
Yu et al. [57], 2012 and USA	To study non‐cancer related mortality rates over time and examine the possible causes for several major death in patients with oral cavity and oropharyngeal cancer.	Cohort study	Suicide completion	32,487 patients across four cohorts.	In the first year after diagnosis of oral cavity and oropharyngeal cancer, suicide was reported only among male patients. During the 3 years after oral cavity and oropharyngeal cancer diagnosis, no suicide deaths occurred among female patients; however, 32 suicides occurred among male patients. Trends for increased risk of suicide were seen among men aged 55–64 years who never married or were divorced, separated, or widowed; had advanced tumour stages; and did not receive treatment.	High
Zaorsky et al. [51], 2019 and USA	To identify cancer patients at higher risk of suicide compared to the general population and other cancer patients	Cohort study	Suicide completion	8,651,569 cancer patients, of which 13,311 died by suicide (81 were patients with a head and neck cancer diagnosis)	Of the 81 suicides that were patients with a head and neck cancer diagnosis, 74 were male and 7 female. The rate of suicide was greatest within the first year of diagnosis, which was reduced between 1 and 5 years and even less after 5‐years post diagnosis.	High

The results will be presented in two parts. The first part will present the findings from the 22 studies that explored suicide completion in patients with head and neck cancer, which includes a meta‐analysis. The second part will provide the narrative synthesis of the studies that explored suicidal ideation in patients with head and neck cancer.

### 3.2 Suicide Completion in Patients With Head and Neck Cancer

Twenty‐two studies are reflective of patients with head and neck cancer who died by suicide. Across 13 of the studies, there were a total of 2,004,476 patients with head and neck cancer, of which 6484 died by suicide [4, 7, 8, 9, 18, 33, 34, 39, 41, 42, 49, 62, 63]. The other nine studies did not report how many patients had a head and neck cancer diagnosis within the sample [43, 44, 51, 52, 53, 54, 55, 56, 57]. 21 of the studies are population‐based cohort studies [7, 8, 9, 18, 33, 34, 39, 41, 42, 43, 44, 49, 51, 52, 53, 54, 55, 56, 57, 62, 63] and one a cross‐sectional study [4]. The studies were conducted in USA [4, 7, 8, 9, 33, 41, 43, 51, 54, 57, 63], Canada [62], Norway [52], Estonia [53], Austria [55], South Korea [9, 34], India [18] Taiwan [42], UK [39], Poland [44], and Lithuania [56]. There was one study conducted in the USA and China [49]. While all 22 studies reported on large population‐based datasets that aids our understanding of the risk factors for suicide completion in patients with head and neck cancer, there was no available data reporting on how suicide could be prevented within this population.

#### 3.2.1 Suicide Completion in Head and Neck Cancer

**FIGURE 2:**
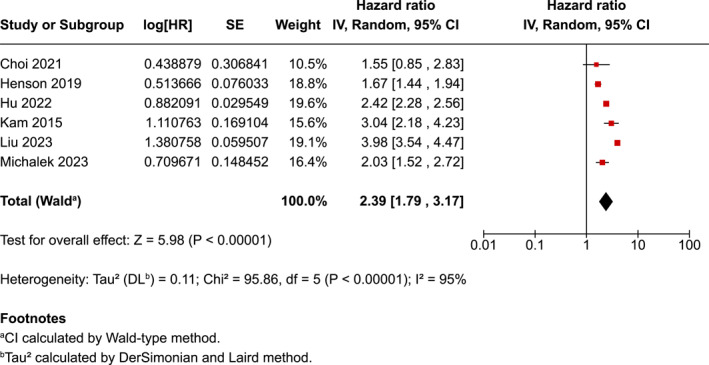
Forest plot for head and neck cancer and suicide completion.

The overall pooled hazard ratio [HR] risk for suicide completion in head and neck cancer patients was 2.39 (95% CI 1.79–3.17) (Figure 2).

#### 3.2.2 Suicide Completion in Head and Neck Cancer and Sex

**FIGURE 3:**
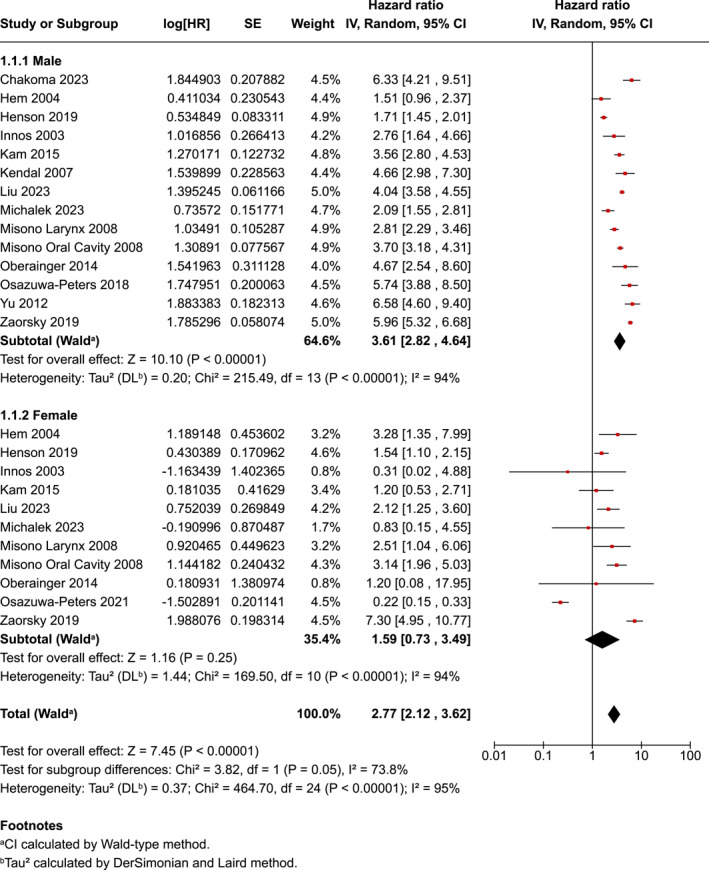
Forest plot for head and neck cancer completion and sex.

Sex was dichotomised in studies as male and female. When sex was compared, the HR risk was higher for males at 3.61 (95% CI 2.82–4.64) compared to females 1.59 (95% CI 0.73–3.49) with a between‐group difference showing a statistically significant difference (*p* = 0.05). There was evidence of considerable heterogeneity in the two sub‐groups of sex (*p* < 0.001); *I* ^2^ = 95%) (Figure 3).

#### 3.2.3 Suicide Completion in Head and Neck Cancer and Age

**FIGURE 4:**
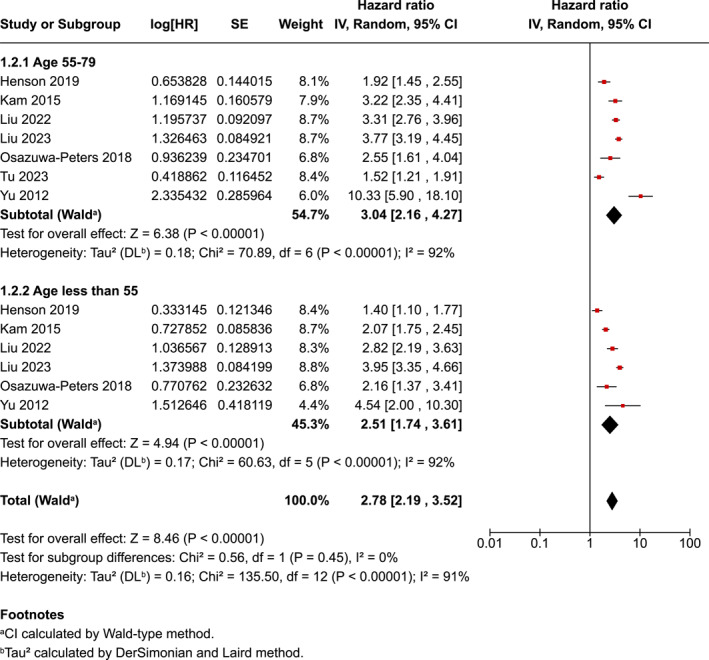
Forest plot for head and neck cancer completion and age.

Age at diagnosis had two subgroups, with the 55–79 years subgroup having a slighter higher risk of suicide (HR ‐ 3.04, 95% CI 2.16–4.27) than the under 55 years (HR−2.51 95% CI 1.74–3.61). This subgroup difference was not reported as statistically significant (*p* = 0.45) (Figure 4). There was evidence of considerable heterogeneity in the two age sub‐groups (*p* < 0.001); *I* ^2^ = 91%).

#### 3.2.4 Suicide Completion in Head and Neck Cancer and Time Since Diagnosis

**FIGURE 5:**
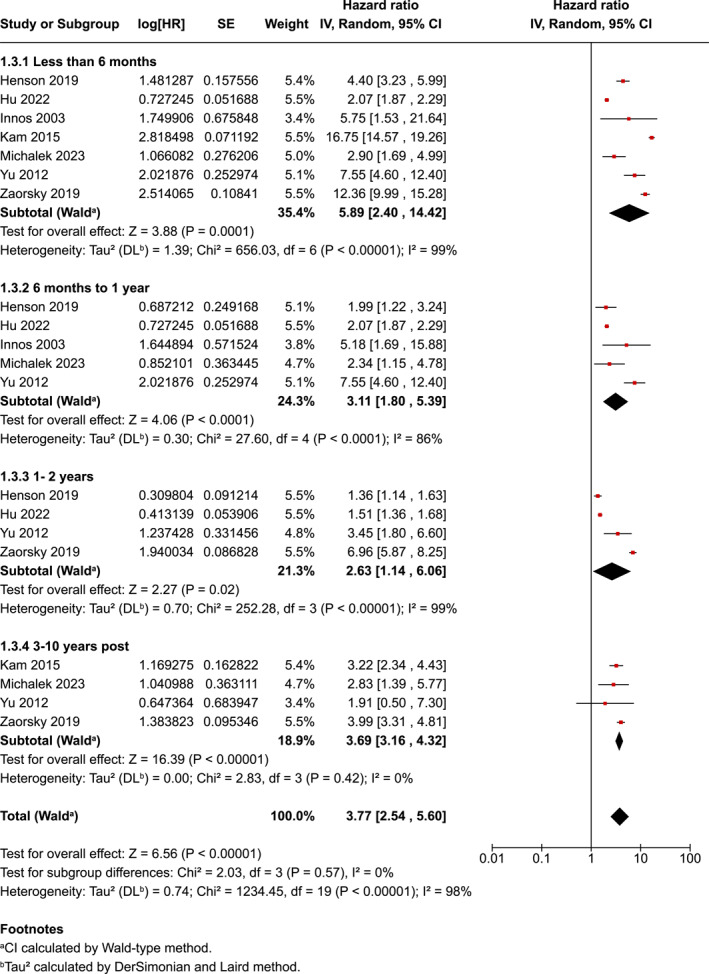
Forest plot for head and neck cancer completion and time since diagnosis.

The time since diagnosis group as a pooled risk was HR−3.77 (95% CI 2.54–5.60) with the highest pooled risk being within 6‐month from diagnosis (HR−5.89, 95% CI 2.40–14.42). The risk then reduced between 6 and less than 12‐month (HR−3.11, 95% CI 1.80–5.39), and was even less at 1–2 years post‐diagnosis (HR−2.63, 95% CI 1.14–6.06). However, between 3 and 10 years post‐diagnosis the risk increased again, being the second highest after the 0–6 months sub‐group (HR−3.69, 95% CI 3.16–4.32). The test for subgroup differences was not statistically significant (*p* = 0.57) (Figure 5). There was evidence of considerable heterogeneity in all sub‐groups of time since diagnosis (*p* < 0.001); *I* ^2^ = 98%).

#### 3.2.5 Suicide Completion in Head and Neck Cancer and Marital Status

**FIGURE 6:**
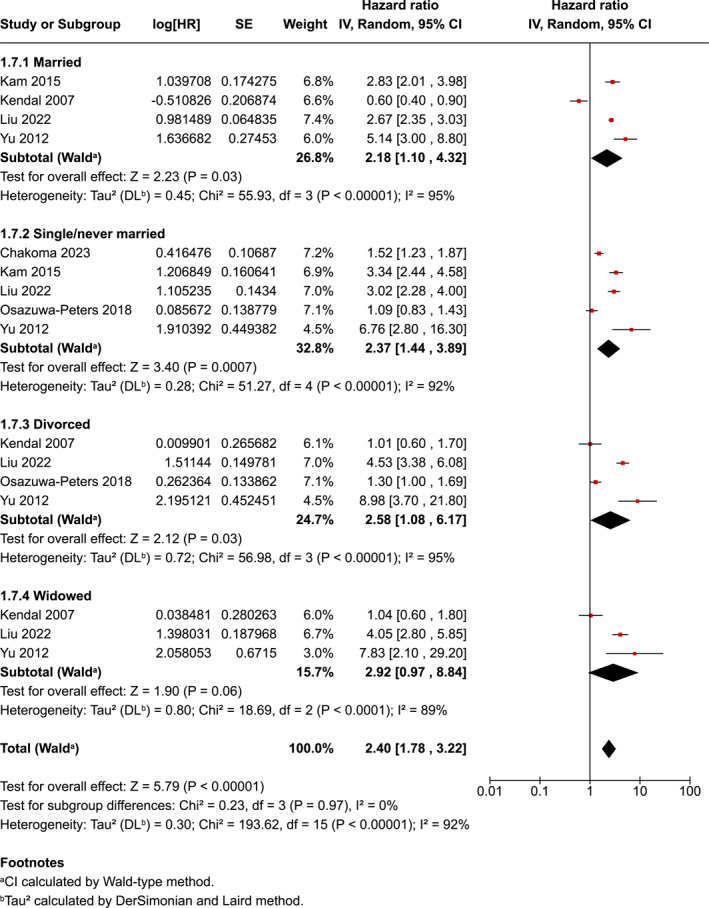
Forest plot for head and neck cancer completion and marital status.

For marital status, the overall pooled risk was 2.40 (95% CI 1.78–3.22) with the widowed group pooled risk higher (HR−2.92, 95% CI 0.97–8.84), followed by divorced (HR−2.58, 95% CI 1.08–6.17], single/never married (HR−2.37 95% CI 1.44–3.89) and married (HR−2.18, 95% CI 1.10–4.32). However, the test for subgroup difference was not significant (*p* = 0.97) (Figure 6). There was evidence of considerable heterogeneity in the four subgroups of marital status (*p* < 0.001); *I* ^2^ = 92%).

### 3.3 Narrative Synthesis: Suicidal Ideation in Patients With Head and Neck Cancer

A narrative synthesis has been conducted on the 14 studies reflective of patients with head and neck cancer who either reported experiences of suicidal ideation, or were identified as having suicidal ideation following inclusion to the study. Of the 14 studies, four were population‐based cohort studies [45, 46, 48, 58]; four were longitudinal studies [38, 39, 45, 60]; two were cross‐sectional studies [36, 47]; three were case studies [40, 59, 61]; and one was a pilot intervention study [50]. The studies were conducted in India [61], USA [46, 59], Poland [40], Japan [58], Taiwan [35, 36, 48], China [47], Canada [37, 38, 60], and UK [50]. Findings are discussed under three themes: (1) importance of identifying and reporting on suicidal ideation in head and neck cancer, (2) identifying the risk factors for suicidal ideation in head and neck cancer, and (3) supportive interventions for patients at risk of suicidal ideation in head and neck cancer.

### 3.4 Theme One: Importance of Identifying and Reporting on Suicidal Ideation in Head and Neck Cancer

Across the three case studies, it appeared thoughts of suicide were first identified and reported to a healthcare professional [40, 59, 61]. This included the patient directly expressing suicidal thoughts to the medical doctor [40], the patient informing the medical professional of a suicide attempt [61], or the patient being described as suicidal following capacity assessment by a psychiatrist [59]. In another study, psychiatric consultation was facilitated for patients with head and neck cancer with poor physical and psychosocial status, including those with poor family support, low socioeconomic status, and severe oral mucositis [35]. Consequently, suicidal ideation was identified after a single detailed psychiatric interviewing by an experienced psychiatrist [35].

Some of the studies identified suicidal ideation in individuals with head and neck cancer using a myriad of validated survey instruments and single‐item questions. This included Beck and Steer's [64] Scale of Suicidal Ideation [38, 60], Barkham and colleagues [65] CORE‐10 screening tool [50], Zhou's [66] Cancer Suicide Risk Scale [47]. The most commonly reported assessment approach was a structured clinical interviews based on the DSM‐IV, facilitated by an experienced psychologist [37, 38, 58, 60]. Similarly, another study identified suicidal ideation in individuals with head and neck cancer who answered ‘yes’ to the question ‘life is no longer worth living’ or ‘I would rather not be alive’ from the DSM‐V diagnoses that are indicative of suicidal ideation [36]. Other population‐based cohort studies identified suicidal ideation or reported suicide attempts in patients with head and neck cancer through national databases that recorded ICD‐9‐CM or ICD‐10 codes [46, 48] or suicide‐related activity [45].

### 3.5 Theme Two: Identifying the Risk Factors for Suicidal Ideation in Head and Neck Cancer

Often, the studies highlighted suicidal ideation was more apparent in males, compared to females with head and neck cancer [35, 48, 58]. Of note, all the case studies were reflective of three men with a head and neck cancer diagnosis, who expressed thoughts of self‐harm or suicide [40, 59, 61]. Hypopharyngeal was identified as a risk factor for suicidal ideation [35, 47]. A recent cohort study in the for patients with head and neck cancer related to functional challenges and suboptimal symptom management to include uncontrolled pain [59, 61]. This included burning sensations as a consequence of radiotherapy [61], excessive saliva [59], bleeding [59], fatigue [59], and difficulties in communicating due to dysarthria [59]. Other consequences of treatment compounding thoughts of suicide were altered eating and drinking, associated with restricted mouth opening and alteration of taste [59, 61]. Similar findings were reported in one of the longitudinal studies, which identified pain and speech impairments as factors associated with increased risk of suicidal ideation [60].

There was inconsistency across the studies in the reporting of treatments received by patients with head and neck cancer and their associated risk for suicidal ideation. While one study noted patients undergoing chemotherapy were at an increased risk of a suicide attempt [48], another study reported higher risks for suicidal ideation head and neck cancer in patients undergoing targeted therapy such as cetuximab [47]. It is unclear from these studies whether the inference related to the treatment modality or the advanced disease status.

Despite the differences in reporting in the studies, there is disparity surrounding when a patient with head and neck cancer is at an increased risk of suicidal ideation. One study highlighted suicidal ideation was greatest within 6‐month of diagnosis, which decreased around 6–11 months, and less again after 1‐year post diagnosis [60]. Similarly, Sun and colleagues [47] highlighted a greater risk of suicidal ideation in patients with head and neck cancer to be within the first 2‐years of diagnosis, a decrease between 2–5 years, and a slight increase in suicidal ideation in patients 5‐year post diagnosis. Separately, it is worth noting that Nugent and colleagues [45] reported that over‐half of suicidal attempts within their cohort occurred after 2‐years post‐diagnosis, with only one‐quarter occurring within 6‐months of diagnosis.

Other factors associated with an increased risk of suicidal ideation that were only reported in one study included a previous cancer diagnosis [35], high demoralisation [36], low income [47], patients aged over 60 [46], and patients with alcohol or nicotine dependence [46]. Of note, the patients in the three case studies were aged between 43 and 55 [40, 59, 61]. Patients with HPV‐negative cancers were reported as more likely to have lifetime pre‐cancer suicidal ideation than patients who were HPV‐positive [38]. Furthermore, patients with body image concerns in the immediate post‐treatment phase were associated with suicidal ideation [37].

### 3.6 Theme Three: Supportive Interventions for Patients at Risk of Suicidal Ideation in Head and Neck Cancer

The case studies highlighted the necessary role of health and social care professionals to address the immediate care needs of patients with head and neck cancer, as a key mechanism to supporting individuals with suicidal thoughts [40, 59, 61]. This included ensuring adequate pain relief was prescribed and available [59, 61], which was especially important when the person was nearing end of life [59]. Also, the provision of education on how to manage symptoms [61]; and help with obtaining financial resources due to no insurance or inadequate income [59]. On occasions, the patients in the case studies did not attend follow‐up appointments [59, 61]. While it was not clear if the patients were at higher risk of suicidal ideation when appointments were missed; nonetheless, the case studies highlighted the necessity of health and social care professionals proactively following up in these situations [59, 61]. This was considered important to identify the individual's care priorities [59, 61] and promote a sense of feeling cared for by the healthcare system [59]. Often, it was considered that during missed appointments, the patients were experiencing an increased intensity of pain and symptoms as a consequence of their head and neck cancer [59, 61].

One study reported reduced suicidal ideation and suicide attempts in patients at end of life with head and neck cancer when referrals were made earlier to palliative care [45]. Also, if a referral was made to a specialist emotional support service, such as psychology, it was considered appropriate to explore if medications needed to be prescribed while waiting for initial appointment [40]. Across other studies, referrals were made to psychology for various reasons, including having a complex mental health history [40], or reaching referral criteria within a study protocol [60]. However, two case studies considered that a referral to psychology was unnecessary as the person's suicidal ideation or attempt was due to inadequate pain and symptom management [59, 61].

While the availability of social and familial support varied for patients in the studies [59, 61], it was not clear what role social support had in managing suicide ideation. However, a suicide attempt was reported in the case study where the person was noted to have a supportive family network [61].

It was identified in the studies that ongoing assessment of suicidal thoughts and support between health and social care professionals and the patient was considered appropriate to manage thoughts of suicide and self‐harm [35, 40, 59, 61]. Follow‐up and supportive care from health and social care professionals with the patients varied across the studies. One case study outlined bi‐weekly visits with a physician and a social worker for reassessment of the person's pain, suicidality, substance abuse and use of prescribed medications [59]. It appeared having continuity of care between the patient and same physician and social worker was helpful for establishing trusting relationships and to express and manage concerns regarding suicidal ideation [59].

When considering types of supportive interventions, one case study involved daily counselling for the person's mental functioning while being treated in the acute hospital setting [61]. It was not clear from the case study who was providing the daily counselling. Upon discharge, the person was followed‐up after 2‐weeks and was reported as ‘more functional’ with no concerns identified for suicidal ideation as pain and symptoms were controlled [61]. Another case study involved psychotherapy, which consisted of daily 45‐min sessions of a combination of verbal therapy, mindfulness and slow breathwork with a psychotherapist during the 7‐week course of radiotherapy [40]. Following this intervention, symptoms of PTSD were described as less intense for the patient, who was reported as ‘healthy’ 1 year following treatment [40].

Within the pilot intervention study to determine the feasibility and effectiveness of an intervention, 19 patients with head and neck cancer were offered up to six appointments with an oncology nurse who were identified as having high level of distress pre‐treatment using the CORE‐10 [50]. The intervention comprised of three key components: (1) psychiatric assessment exploring the patient's history of depressive symptoms and presenting concerns and issues, (2) conversations with the patient about their emotional state, and (3) liaising with multi‐disciplinary professionals such as medication reviews by primary care professionals, or for additional psychosocial support provided by cancer charity organisations [50]. The oncology nurse required 3‐months training in a psychology department shadowing a psychiatrist and psychiatric nurse, 3 months of seeing patients under supervision, and completion of a 3‐day empathy course prior to facilitating the intervention [50]. Depressive symptoms and suicidal ideation were reported as reduced following partial and full completion of the six sessions as measured using the CORE‐10 screening tool [50]. There were no other interventional studies identified within the literature.

## 4 Discussion

### 4.1 Summary of Findings

It was identified that being male was statistically significant (*p* = 0.05) as the highest risk factor for suicide completion in patients with head and neck cancer. Although not statistically significant, a higher risk of suicide completion was observed in patients aged 55–79 (in comparison to under 55 years), within the first 6‐months of diagnosis, patients who were widowed or had a head and neck cancer diagnosis of the hypopharynx.

Often, suicidal ideation was more apparent in males compared to females with head and neck cancer. Suboptimal pain and symptom management appeared related to a higher risk of suicidal ideation in patients with head and neck cancer. It seemed that a therapeutic and supportive relationship between health and social care professionals and patients with head and neck cancer was helpful in managing experiences of suicidal ideation.

There was inconclusive evidence regarding risk factors for suicidal completions and suicide ideation in patients with head and neck cancer regarding cancer staging, treatment options, substance misuse, financial toxicity and psychological distress.

### 4.2 Discussion of Findings

From a biological perspective, males were at increased risk of suicide completion and suicidal ideation compared to females. While the World Health Organisation identifies females at higher risk of suicidal ideation, males are 2.3 times more likely to die by suicide than females [67]. Increased suicide rates in males could be explained by the reality that males are two times more likely to develop head and neck cancer compared to females [68]. Considering male as a gender construct, certain societal, cultural or self‐expectations may place males at higher risk of suicide if they view themselves as a failure, feel like a burden to others, or less able to provide for their family [69, 70]. Nonetheless, these findings suggest that male patients with head and neck cancer are particularly vulnerable to suicidality compared to females.

Compared to other head and neck cancer tumour subsites, hypopharyngeal cancer is often metastasised at the time of diagnosis [71], necessitating many patients to undergo multi‐modality therapy to include surgery and chemo‐radiotherapy [72]. Despite advances in diagnostic and treatment options [73, 74], the 5‐year survival rate for patients with hypopharyngeal cancer remains low at approximately 30% [75]. Many patients experience a reduction in hypopharyngeal function following treatment which significantly impacts on factors related to quality of life such as altered eating and drinking, communication abilities, intimacy and sexuality [73, 76, 77, 78]. The reduction or absence of these important aspects of everyday life has major psychosocial impacts, which often negatively contribute to a person's wellness, coping and overall functioning [77]. Unsurprisingly, a reduction in psychosocial wellbeing is correlational to suicidal ideation and suicide completion [79].

Receiving a head and neck cancer diagnosis is often associated with fatigue, reduced coping and a diminished sense of self, often contributing to distress [14, 80, 81, 82]. Head and neck cancer‐related distress is commonly multi‐faceted and often a function of the diagnosis itself, the presence of burdensome disease, treatment sequelae, and declines in health‐related quality of life [83, 84]. Despite patients desire and pursuit to regain normal everyday functioning and aesthetics, many patients with head and neck cancer fail 1‐year post‐treatment [85, 86]. Consequently, this can lead to social and relational disruptions, considered as a ‘loss’ for patients and carers [15, 16, 85, 86, 87, 88, 89]. Alongside this, and akin to other tumour groups, patients with head and neck cancer experience fear of recurrence [90]. This is often associated with reduced psychological coping [91] and interestingly is frequently the chief post‐treatment concern for patients with head and neck cancer [87, 92], affecting from half to more than three‐fourths of patients [93]. These factors may help to understand why risk of suicidality is prevalent in patients with head and neck cancer.

Furthermore, patients experiencing persistent depressive effect are more likely to report worse head and neck specific health‐related quality of life outcomes [3]. Arguably, living long‐term with the consequences of treatment, or experiencing new burdensome late effects of treatment, may explain why there is an increase in suicidal ideation and suicide completion 3‐years post‐diagnosis [94, 95]. It could be suggested that there are patients who are ‘weary’ of living with the consequences of head and neck cancer treatment at this timepoint [36]. Additionally, findings within this review indicate that older people and those who are widowed are at greater risk of suicide completion in head and neck cancer. A recent systematic review on quality of life for older people with head and neck cancer reported that post‐treatment, older people were more reliant on avoidant coping strategies in comparison to younger patients who were more likely to use active positive coping strategies [96]. Later life is a period of major transition [6] and can often be a period where older people struggle to find meaning for late‐life living [97]. It could be argued that due to the absence of previously available support networks (i.e. spouse/partner) in these challenging and stressful situations contributes to a sense of ‘*giving up’* or feeling *‘life isn't worth living’* [97, 98, 99].

### 4.3 Clinical Implications

More often, patients are likely to present to health and social care services with physical challenges rather than psychosocial issues such as thoughts of suicide [100]. Health and social care professionals are ideally placed to identify, assess, support and follow‐up with patients with head and neck cancer regarding suicidal ideation [11, 101, 102]. Despite this, clinicians have reported a lack of training and awareness in differentiating suicidality from mental health distress, lack of time with patients during clinical appointments, fear of asking and ‘opening a can of worms’, a lack of referral pathways, lack of access to timely psychological interventions and lack of coping resources for both patients and clinicians [11, 103]. Alongside this, it is apparent from this review that there is no agreement on the appropriate method on how best professionals should identify patients at risk of suicide ideation. Also, there is a lack of evidence‐based interventions for the management of suicide ideation in this population to prevent suicide completion [102]. There is a need for stepped‐care pathways for management of suicidal ideation in head and neck cancer [104] which should include appropriate psychotherapeutic and/or pharmacological treatment [3]. Furthermore, it is pertinent that health and social care professionals are equipped with adequate skills and training to be able to assess, identify and manage suicide ideation within this population.

It appears the provision of timely and adequate pain and symptom management can help mediate suicidal ideation in patients with head and neck cancer [59, 105]. In the acute post‐treatment phase, there is a need to ensure the physical, social and emotional symptoms of patients with head and neck cancer are understood and addressed by health and social care professionals [106]. There has been some growth in the publication of post‐treatment psychosocial interventions for patients with head and neck cancer [107]. However, there remains a paucity of high quality, theoretically based, randomised controlled trials examining the efficacy of such interventions, particularly compared to other cancer literature [108].

A recent randomised controlled trial reported the beneficial social‐emotional effect of integrating the ‘Patient Concerns Inventory’ [109] as a communication prompt aid in routine follow‐up care to identify key concerns and priorities related to a patient's physical and functional wellbeing, psychosocial aspects and treatment related challenges [110]. Enabling patients to systematically identify symptoms and concerns, promotes timely access for symptom management and psychological services [111], in contrast to a system reliant on the initiative of the healthcare providers which commonly equates to patients being glibly asked ‘*how are you?*’. In these situations, patients may not honestly share with clinicians how they are feeling for a fear of ‘letting the professional down’ who invested on ‘saving their life’ [112]. A Patient Concerns Inventory would be especially important within the first 6‐months of diagnosis and in advance of discharge from routine follow‐up when the risks for suicide ideation and suicide completion are considered greatest. The benefits of using a ‘Patient Concerns Inventory’ in routine clinical care has enabled patients to articulate their thoughts, especially those from low socioeconomic backgrounds, which represents a huge preponderance of the head and neck cancer population [113].

### 4.4 Implication of Findings to Policy

Multi‐agency suicide prevention partnerships are required to ensure coordinated processes are in place to identify and provide opportunities for individuals to access support who are at risk of, or impacted by suicide [114]. However, there are only 38 countries that report having a national suicide prevention strategy [16]. Given the global incidence of suicide in patients with head and neck cancer being three‐times of the general population [8], policy must acknowledge the key issues facing patients with head and neck cancer. This includes nuances that are often absent from suicide prevention strategies such as being older (60+) [6]. A public health approach to talking about thoughts of suicide and cancer could help to promote conversations about suicide ideation for people with head and neck, and reduce the risk of suicide completion within this population [6].

### 4.5 Future Research

It is estimated that 77% of global suicides occur in low‐and‐middle‐income countries [115]. However, only two studies in this review [18, 61] were conducted within a low‐and‐middle income country [116]. Alongside this, head and neck cancer is a tumour group with a high prevalence of patients from lower socioeconomic backgrounds [113, 117]. To address inequalities in suicide prevention policy, and promote better access to services and support, there is a need to better understand the experiences and needs of this population, who are at greater risk of suicidality than any other tumour group [8]. This includes factors related to quality of life, the impact and risk of substance misuse in suicidal ideation [118], and the provision of supportive care from health and social care professionals.

Many of the cohort study databases on suicide completion were representative of patients diagnosed with head and neck cancer and died by suicide between 1970 and 2010. Head and neck cancer has been a changing landscape with a shift and trend in diagnoses over the last 20‐year from older males with a significant history of alcohol and smoking dependency to a population cohort with a greater spread of representation of being female and younger [119]. Further research is required to explore the perceived risk and experiences of suicidality in head and neck cancer within these populations.

### 4.6 Strengths and Limitations

A rigorous and transparent approach was followed in the conduct and reporting of this systematic review. Large data sets allowed a meta‐analysis to be conducted on several risk factors for suicide completion in head and neck cancer. Due to missing, or inconsistencies in reporting of data related to tumour site, treatment type, cancer staging, HPV status and psychiatric symptomatology it was not possible to conduct a meta‐analysis on these factors. Similarly, due to insufficient or inconsistencies in reporting, a meta‐analysis could not be conducted on risk factors for suicide ideation in head and neck cancer. Nonetheless, they have been reported descriptively to portray a picture of this phenomena of interest. It is possible studies not published in the English language are excluded from this systematic review. It is unclear what impact staging has on suicidality in head and neck cancer. The inclusion of patients with lived experience of head and neck cancer was helpful in shaping the clinical recommendations that were perceived to be most pertinent for clinical practice. Supportive networks such as carers have an instrumental role in providing emotional support during the cancer experience [120] and are often a mediating factor in preventing death by suicide and promoting well‐being [121]. The caregiving role can often be burdensome for carers in head and neck cancer [120]. The protocol of this review further aimed to explore the experience, challenges, needs of carers but identified no studies, necessitating for further research.

## 5 Conclusion

Literature highlights the incidence of suicidality in patients in head and neck cancer is thrice of the general population. Males with head and neck cancer were statistically at greater risk of death by suicide. Health and social care professionals are pivotal to identifying, assessing, supporting and following‐up patients with head and neck cancer experiencing suicidal ideation. Within clinical practice, clear pathways, optimal symptom management and timely access to psychological interventions are required to support patients with head and neck cancer experiencing suicidality and mitigate rates of suicide completion. Further research is necessary to investigate the detailed mechanisms and elucidate the causal pathways that link risks to suicidality in head and neck cancer in patients and carers experiencing suicidality.

## Author Contributions

JRH and CJS conceptualised the study. JRH obtained funding from the Western Health and Social Care Trust. JRH and CJS developed the study protocol. The search strategy was developed by JRH, CJS and KMcC. Database searching was completed by JRH, JA and KMcC. Grey literature searches were completed by JRH and CJS. Data extraction was completed by JRH and KMcC. Quality assessment was completed by AMcK, JRH and CJS. Meta‐analysis was completed by KMcC. Narrative synthesis was completed by JRH and CJS. Coordination of PPI activities and integration of their voices to the review were led by CJS and JRH. Manuscript preparation was led by JRH and CJS. All authors approved the final version.

## Conflicts of Interest

The authors declare no conflicts of interest.

## Data Availability

Data is available upon reasonable request from the first author [JRH].

## Appendix 1 PRESS tool

**Table:**

Question	Response	Question	Response
**Translation of the research questions**		Are subheadings attached to subheading headings?	
Does the search strategy match the research question/PICO?	**Y**	Are floating subheadings and terms in free text used for each concept?	**Y**
Are the search concepts clear?	**Y**	**Text word searching**	
Are there too many or too few PICO elements included?	**N**	Does the search include all spelling variants in free text (eg, UK vs. US spelling)?	**Y**
Are the search concepts too narrow or too broad?	**N**	Does the search include all synonyms or antonyms (eg. opposites)?	**Y**
Does the search retrieve too many or too few records?	**N**	Does the search capture relevant truncation (ie, is truncation at the correct place)?	**Y**
Are unconventional or complex strategies explained?	**Y**	Is the truncation too broad or too narrow?	**N**
**Boolean and proximity operators**		Are acronyms or abbreviations used appropriately? Do they capture irrelevant material? Are the full terms also included?	**Y**
Are Boolean or proximity operators used correctly?	**Y**	Are the keywords specific enough or too broad?	**Ok**
Is the use of nesting with brackets appropriate and effective and effective for the search?	**Y**	Have the appropriate fields been searched?	
If NOT is used, is this likely to result in any unintended exclusions?	**N/A**	Should any long strings be broken into several shorter search statements?	**N**
Could precision be improved, by using proximity operators (eg, adjacent, near, within) or phrase searching instead of AND?	**N**	**Spelling, syntax, and line numbers**	
Is the width of proximity operators suitable (eg, might adj5 pick up more variants than adj2)?	**Y**	Are there spelling errors?	**Y**
**Subject headings**		Are there any errors in system syntax?	**N**
Are the subject heading relevant?	**Y**	Are there incorrect line combinations or orphan lined?	**N**
Are any relevant subject headings missing; for example, previous index terms?	**N**	**Limits and filters**	
Are subject headings exploded where necessary and vice versa?	**Y**	Are all limits and filters used appropriately and relevant given the research question?	**Y**
Are major headings (“starring” or restrict to focus) used? If so, is there adequate justification?	**Y**	Are all limits and filters used appropriately and relevant for the database?	**Y**
Are subheadings missing?	**N**	Are any potentially helpful limits or filters missing?	**N**
Are any subject headings too broad or too narrow?	**N**	Are sources cited for the filters used?	**N/A**

## Appendix 2 Quality Assessment

### Critical Appraisal Skills Programme (CASP) Quality Assessment Tool for Cohort Studies

**Table:**

Paper	Did the study address a clearly focussed issue?	Was the cohort recruited in an acceptable way?	Was the exposure accurately measured to minimise bias?	Was the outcome accurately measured to minimise bias?	Have the authors identified all important confounding factors?	Have the authors taken account of the confounding factors in the design and/or analysis?	Was the follow up of subjects complete enough?	Was the follow up of subjects long enough?	Do you believe the results?	Can the results be applied to the local population?	Do the results of this study fit with other available evidence?	Quality assessment ‐ GRADE
Akechi et al. [58]	Yes	Yes	Yes	Yes	Yes	Yes	Can't tell	Yes	Yes	Yes	Yes	90% ‐ High
Chakoma et al. [33]	Yes	Yes	Yes	Yes	Yes	Yes	Yes	Yes	Yes	Yes	Yes	100% ‐ High
Chang et al. [35]	Yes	Yes	Yes	Yes	Yes	Yes	Yes	Yes	Yes	Yes	Yes	100% ‐ High
Choi and Park [9]	Yes	Yes	Yes	Yes	Yes	Yes	Yes	Yes	Yes	Yes	Yes	100% ‐ High
Choi et al. [34]	Yes	Yes	Yes	Yes	Yes	Yes	Yes	Yes	Yes	Yes	Yes	100% ‐ High
Hem et al. [52]	Yes	Yes	Yes	Yes	Yes	Yes	Yes	Yes	Yes	Yes	Yes	100% ‐ High
Henry et al. [60]	Yes	Yes	Yes	Yes	Yes	Yes	Yes	Yes	Yes	Yes	Yes	100% ‐ High
Henry et al. [37]	Yes	Yes	Yes	Yes	Yes	Yes	Yes	No	Yes	Yes	Yes	90% ‐ High
Henry et al. [38]	Yes	Yes	Yes	Yes	Yes	Yes	Yes	No	Yes	Yes	Yes	90% ‐ High
Henson et al. [39]	Yes	Yes	Yes	Can't tell	Yes	Yes	Yes	Yes	Yes	Yes	Yes	90% ‐ High
Hu et al. [41]	Yes	Yes	Yes	Yes	Yes	Yes	Yes	Yes	Yes	Yes	Yes	100% ‐ High
Innos et al. [53]	Yes	Yes	Yes	Yes	Yes	Yes	Yes	Yes	Yes	Yes	Yes	100% ‐ High
Kam et al. [8]	Yes	Yes	Yes	Yes	Yes	Yes	Yes	Yes	Yes	Yes	Yes	100% ‐ High
Kendal [62]	Yes	Yes	Yes	Yes	Yes	Yes	Yes	Yes	Yes	Yes	Yes	100% ‐ High
Liu et al. [43]	Yes	Yes	Yes	Yes	Yes	Yes	Yes	Yes	Yes	Yes	Yes	100% ‐ High
Liu et al. [42]	Yes	Yes	Yes	Yes	Yes	Yes	Yes	Yes	Yes	Yes	Yes	100% ‐ High
Michalek et al. [44]	Yes	Yes	Yes	Yes	Yes	Yes	Yes	Yes	Yes	Yes	Yes	100% ‐ High
Misono et al. [63]	Yes	Yes	Yes	Yes	Yes	Yes	Yes	Yes	Yes	Yes	Yes	100% ‐ High
Nasseri et al^.54^	Yes	Yes	Yes	Yes	Yes	Yes	Yes	Yes	Yes	Yes	Yes	100% ‐ High
Nugent et al. [45]	Yes	Yes	No	No	Yes	Yes	Yes	Yes	Yes	Yes	Yes	81% ‐ High
Oberainger et al. [55]	Yes	Yes	Yes	Yes	Yes	Yes	Yes	Yes	Yes	Yes	Yes	100% ‐ High
Osazuwa‐Peters et al. [7]	Yes	Yes	Yes	Yes	Yes	Yes	Yes	Yes	Yes	Yes	Yes	100% ‐ High
Smailyte et al. [56]	Yes	Yes	Can't tell	Yes	Yes	Yes	Yes	Yes	Yes	Yes	Yes	90% ‐ High
Stanbouly et al. [46]	Yes	Yes	No	No	Yes	Yes	No	No	Yes	Yes	Yes	63% ‐Moderate
Sun et al. [48]	Yes	Yes	No	Yes	Yes	Yes	Yes	Yes	Yes	Yes	Yes	90% ‐ High
Thavarajah et al. [18]	Yes	Yes	No	Yes	Yes	Yes	Yes	Yes	Yes	Yes	Yes	90% ‐ High
Tu et al. [49]	Yes	Yes	Yes	Yes	Yes	Yes	Yes	Yes	Yes	Yes	Yes	100% ‐ High
Yu et al. [57]	Yes	Yes	Yes	Yes	Yes	Yes	Yes	Yes	Yes	Yes	Yes	100% ‐ High
Zarosky et al. [51]	Yes	Yes	Yes	Yes	Yes	Yes	Yes	Yes	Yes	Yes	Yes	100% ‐ High

### JBI Critical Appraisal Checklist for Analytical Cross‐Sectional Studies

**Table:**

Paper	Were the criteria for inclusion in the sample clearly defined?	Were the study subjects and the setting described in detail?	Was the exposure measured in a valid and reliable way?	Were objective, standard criteria used for measurement of the condition?	Were confounding factors identified?	Were strategies to deal with confounding factors stated?	Were the outcomes measured in a valid and reliable way?	Was appropriate statistical analysis used?	Overall appraisal?	Quality assessment ‐ GRADE
Chang et al. [36]	Yes	Yes	Yes	Yes	Yes	Unclear	Yes	Yes	Include	87.5% ‐ High
Osazuwa‐Peters et al. [4]	Yes	Yes	Yes	Yes	N/A	N/A	Yes	Yes	Include	75% ‐ Moderate
Sun et al. [47]	Yes	Yes	Yes	Yes	NA	NA	Yes	Yes	Include	75% ‐ Moderate

### JBI Critical Appraisal Checklist for Quasi‐Experimental Studies

**Table:**

Paper	Is it clear in the study what is the ‘cause’ and what is the ‘effect’	Was there a control group?	Were participants included in any comparisons similar?	Were the participants included in any comparisons receiving similar treatment/care, other than the exposure or intervention of interest	Were there multiple measurement of the outcome, both pre and post the intervention/exposure?	Were the outcomes of participants included in any comparisons measured in the same way?	Were the outcomes measured in a reliable way?	Was follow‐up complete, and if not, were differences between groups in terms of their follow‐up adequately described and analysed?	Was appropriate statistical analysis used?	Overall appraisal	Quality assessment ‐ GRADE
Waltho et al. [50]	Yes	No	No	No	Yes	N/A	Yes	Yes	Yes	Include	55% ‐ Moderate

### JBI Critical Appraisal Checklist for Care Reports

**Table:**

Criteria	Goelitz et al. [59]	Heyda et al. [40]	Kapoor and Bhatnagar [61]
Were patient's demographic characteristics clearly described?	Yes	Yes	Yes
Was the patient's history clearly described and presented as a timeline?	No	Yes	Yes
Was the current clinical condition of the patient on presentation clearly described?	Yes	Yes	Yes
Were diagnostic tests or assessment methods and the results clearly described?	Unclear	Yes	Unclear
Was the intervention(s) or treatment procedure(s) clearly described?	Yes	Yes	Yes
Was the post‐intervention clinical condition clearly described?	Yes	Yes	Unclear
Were adverse events (harms) or unanticipated events identified and described?	Not applicable	Not applicable	Yes
Does the case report provide takeaway lessons?	Yes	Yes	Yes
Overall appraisal	Include	Include	Include
Quality assessment (GRADE)	62.5% ‐ Moderate	87.5% ‐ High	75% ‐ Moderate

## Appendix 3 CERQual of the Three Themes within the Narrative Synthesis

**Table:**

Theme	Studies contributing to this theme	Assessment of methodological limitations	Assessment of relevance	Assessment of coherence	Assessment of adequacy	Overall CERQual assessment of confidence	Explanation of judgement
Screening for and reporting of suicidal ideation in head and neck cancer	Akechi et al. [58] Chang et al. [35] Chang et al. [36] Goelitz et al. [59] Henry et al. [37] Henry et al. [38] Henry et al. [60] Heyda et al. [40] Kapoor and Bhatnagar [61] Nugent et al. [45] Stanbouly et al. [46] Sun et al. [47] Sun et al. [48] Waltho et al. [50]	Minor methodological limitations (all studies had minor methodological limitations)	Minor concerns about relevance (all studies were moderately relevant)	Moderate concerns about coherence (screening and identification of suicidal ideation varied across the studies with no consistency)	Minor concerns about adequacy (all studies offered moderately rich detail about how suicidal ideation was identified)	**Moderate confidence**	This theme was graded as moderate confidence because of minor concerns concerning methodological limitations, relevance and adequacy of the data. Also, due to moderate concerns about the coherence between the studies
Identifying the risk factors for suicide ideation in head and neck cancer	Akechi et al. [58] Chang et al. [35] Chang et al. [36] Goelitz et al. [59] Henry et al. [37] Henry et al. [38] Henry et al. [60] Heyda et al. [40] Kapoor and Bhatnagar [61] Nugent et al. [45] Stanbouly et al. [46] Sun et al. [47] Sun et al. [48] Waltho et al. [50]	Minor methodological limitations (all studies had minor methodological limitations)	Minor concerns about relevance (all studies were moderately relevant)	Moderate concerns about coherence (Inconsistency in risk factors across some studies. Some risk factors were only reported in one study).	Minor concerns about adequacy (all studies offered rich detail about risk factors for suicidal ideation)	**Moderate confidence**	This theme was graded as moderate confidence because of minor concerns concerning methodological limitations, relevance and adequacy of the data. Also, due to moderate concerns about the coherence between the studies
Supportive interventions for patients at risk of suicidal ideation in head and neck cancer	Chang et al. [35] Goelitz et al. [59] Henry et al. [60] Heyda et al. [40] Kapoor and Bhatnagar [61] Nugent et al. [45] Waltho et al. [50]	Minor methodological limitations (all studies had minor methodological limitations)	Moderate concerns about relevance (3 studies were moderately relevant; 4 had partial relevance)	Moderate concerns about coherence (Inconsistency across the studies about how and when best to support patients experiencing suicidal ideation)	Minor concerns (5 studies offered moderately rich data; 2 studies had partial relevance)	**Moderate confidence**	This theme was graded low confidence because of moderate concerns to relevance and coherence

## References

[1] World Health Organization . “Suicide Worldwide in 2019,” accessed: May 7, 2025, https://iris.who.int/bitstream/handle/10665/341728/9789240026643‐eng.pdf.

[2] S. Rafiei , F. Pashazadeh Kan , S. Raoofi , et al. “Global Prevalence of Suicide in Patients With Cancer: A Systematic Review and Meta‐Analysis,” Archives of Suicide Research 28, no. 3 (July 2024): 723–736, 10.1080/13811118.2023.2240870. 37578189

[3] P. Jimenez‐Labaig , C. Aymerich , I. Brana , et al. “A Comprehensive Examination of Mental Health in Patients With Head and Neck Cancer: Systematic Review and Meta‐Analysis,” JNCI Cancer Spectrum 8, no. 3 (June 2024): pkae031, 10.1093/jncics/pkae031. 38702757 PMC11149920

[4] N. Osazuwa‐Peters , J. M. Barnes , S. I. Okafor , et al. “Incidence and Risk of Suicide Among Patients With Head and Neck Cancer in Rural, Urban, and Metropolitan Areas,” JAMA Otolaryngology—Head & Neck Surgery 147, no. 12 (December 2021): 1045–1052, 10.1001/jamaoto.2021.1728. 34297790 PMC8304170

[5] Y. V. Men , T. C. Lam , C. Y. Yeung , and P. S. Yip , “Understanding the Impact of Clinical Characteristics and Healthcare Utilizations on Suicide Among Cancer Sufferers: A Case‐Control Study in Hong Kong,” Lancet Regional Health–Western Pacific 17 (December 2021): 100298, 10.1016/j.lanwpc.2021.100298. 34734204 PMC8551816

[6] T. Hafford‐Letchfield , J. R. Hanna , T. J. Ellmers , et al. “Talking Really Does Matter: Lay Perspectives From Older People on Talking About Suicide in Later Life,” Frontiers in Psychology 13 (November 2022): 1009503, 10.3389/fpsyg.2022.1009503. 36467190 PMC9709258

[7] N. Osazuwa‐Peters , M. C. Simpson , L. Zhao , et al. “Suicide Risk Among Cancer Survivors: Head and Neck versus Other Cancers,” Cancer 124, no. 20 (October 2018): 4072–4079, 10.1002/cncr.31675. 30335190

[8] D. Kam , A. Salib , G. Gorgy , et al. “Incidence of Suicide in Patients With Head and Neck Cancer,” JAMA Otolaryngology—Head & Neck Surgery 141, no. 12 (December 2015): 1075–1081, 10.1001/jamaoto.2015.2480. 26562764

[9] Y. Choi and E. C. Park , “Suicide After Cancer Diagnosis in South Korea: A Population‐Based Cohort Study,” BMJ Open 11, no. 9 (September 2021): e049358, 10.1136/bmjopen-2021-049358. PMC8413965 34475169

[10] Y. S. Jung , D. Lee , K. W. Jung , and H. Cho , “Long‐Term Survivorship and Non‐Cancer Competing Mortality in Head and Neck Cancer: A Nationwide Population‐Based Study in South Korea. Cancer Research and Treatment,” Official Journal of Korean Cancer Association 55, no. 1 (March 2022): 50–60, 10.4143/crt.2021.1086. PMC9873318 35698446

[11] K. Turner , A. M. Stover , D. B. Tometich , et al. “Oncology Providers' and Professionals' Experiences With Suicide Risk Screening Among Patients With Head and Neck Cancer: A Qualitative Study,” JCO Oncology Practice 19, no. 6 (June 2023): e892–e903, 10.1200/op.22.00433. 36395441 PMC10337750

[12] M. D. Mody , J. W. Rocco , S. S. Yom , R. I. Haddad , and N. F. Saba , “Head and Neck Cancer,” Lancet 398, no. 10318 (December 2021): 2289–2299, 10.1016/s0140-6736(21)01550-6. 34562395

[13] L. Millsopp , L. Brandom , G. Humphris , D. Lowe , C. Stat , and S. Rogers , “Facial Appearance After Operations for Oral and Oropharyngeal Cancer: A Comparison of Casenotes and Patient‐Completed Questionnaire,” British Journal of Oral and Maxillofacial Surgery 44, no. 5 (October 2006): 358–363, 10.1016/j.bjoms.2005.07.017. 16236404

[14] H. W. Schutte , F. Heutink , D. J. Wellenstein , et al. “Impact of Time to Diagnosis and Treatment in Head and Neck Cancer: A Systematic Review,” Otolaryngology–Head and Neck Surgery 162, no. 4 (April 2020): 446–457, 10.1177/0194599820906387. 32093572

[15] M. Dornan , C. Semple , and A. Moorhead , “Experiences and Perceptions of Social Eating for Patients Living With and Beyond Head and Neck Cancer: A Qualitative Study,” Supportive Care in Cancer 30, no. 5 (May 2022): 4129–4137, 10.1007/s00520-022-06853-6. 35072791 PMC8785386

[16] World Health Organisation . “Suicide,”2023. Accessed: 25 April 2024, https://www.who.int/news‐room/fact‐sheets/detail/suicide.

[17] J. J. Mann , C. A. Michel , and R. P. Auerbach , “Improving Suicide Prevention through Evidence‐Based Strategies: A Systematic Review,” American Journal of Psychiatry 178, no. 7 (July 2021): 611–624, 10.1176/appi.ajp.2020.20060864. 33596680 PMC9092896

[18] R. Thavarajah , A. A. Mohandoss , E. Joshua , U. K. Rao , and K. Ranganathan , “Is Suicide a Significant Contributor to Mortality in Head and Neck Cancer: A Surveillance, Epidemiology, and End Results Database Study,” Journal of Global Oral Health 1 (July 2018): 37–48, 10.25259/JGOH-13-2018.

[19] M. J. Page , J. E. McKenzie , P. M. Bossuyt , et al. “The PRISMA 2020 Statement: An Updated Guideline for Reporting Systematic Reviews,” BMJ 372 (May 2021): n71, 10.1136/bmj.n71. 33782057 PMC8005924

[20] C. Schardt , M. B. Adams , T. Owens , S. Keitz , and P. Fontelo , “Utilization of the PICO Framework to Improve Searching PubMed for Clinical Questions,” BMC Medical Informatics and Decision Making 7 (December 2007): 1–6, 10.1186/1472-6947-7-16. 17573961 PMC1904193

[21] J. McGowan , M. Sampson , D. M. Salzwedel , E. Cogo , V. Foerster , and C. Lefebvre , “PRESS Peer Review of Electronic Search Strategies: 2015 Guideline Statement,” Journal of Clinical Epidemiology 75 (July 2016): 40–46, 10.1016/j.jclinepi.2016.01.021. 27005575

[22] M. Roerecke and J. Rehm , “Alcohol Use Disorders and Mortality: A Systematic Review and Meta‐Analysis,” Addiction 108, no. 9 (September 2013): 1562–1578, 10.1111/add.12231. 23627868

[23] M. Roerecke and J. Rehm , “Chronic Heavy Drinking and Ischaemic Heart Disease: A Systematic Review and Meta‐Analysis,” Open heart 1, no. 1 (August 2014): e000135, 10.1136/openhrt-2014-000135. 25332827 PMC4189294

[24] R. DerSimonian and N. Laird , “Meta‐Analysis in Clinical Trials,” Controlled Clinical Trials 7, no. 3 (September 1986): 177–188, 10.1016/0197-2456(86)90046-2. 3802833

[25] W. G. Cochran , “The Combination of Estimates From Different Experiments,” Biometrics 10, no. 1 (March 1954): 101–129, 10.2307/3001666.

[26] J. P. Higgins and S. G. Thompson , “Quantifying Heterogeneity in a Meta‐Analysis,” Statistics in Medicine 21, no. 11 (June 2002): 1539–1558, 10.1002/sim.1186. 12111919

[27] J. J. Deeks , J. P. Higgins , and D. G. Altman , and Cochrane Statistical Methods Group . “Analysing Data and Undertaking Meta‐Analyses,” Cochrane Handbook for Systematic Reviews of Interventions 23 (September 2019): 241–284, 10.1002/9781119536604.ch10.

[28] J. Popay , H. Roberts , A. Sowden , et al. “Guidance on the Conduct of Narrative Synthesis in Systematic Reviews,” A Product From the ESRC Methods Programme Version 1, no. 1 (April 2006): b92.

[29] J. Thomas and A. Harden , “Methods for the Thematic Synthesis of Qualitative Research in Systematic Reviews,” BMC Medical Research Methodology 8 (December 2008): 1, 10.1186/1471-2288-8-45. 18616818 PMC2478656

[30] S. Lewin , A. Booth , C. Glenton , et al. “Applying GRADE‐CERQual to Qualitative Evidence Synthesis Findings: Introduction to the Series,” Implementation Science 13, no. 1 (January 2018), 10.1186/s13012-017-0688-3. PMC5791040 29384079

[31] T. H. Barker , J. C. Stone , K. Sears , et al. “The Revised JBI Critical Appraisal Tool for the Assessment of Risk of Bias for Randomized Controlled Trials,” JBI Evidence Synthesis 21, no. 3 (March 2023): 494–506, 10.11124/jbies-22-00430. 36727247

[32] S. Rennick‐Egglestone , K. Morgan , J. Llewellyn‐Beardsley , et al. “Mental Health Recovery Narratives and Their Impact on Recipients: Systematic Review and Narrative Synthesis,” Canadian Journal of Psychiatry 64, no. 10 (October 2019): 669–679, 10.1177/0706743719846108. 31046432 PMC6783672

[33] T. Chakoma , P. K. Moon , O. L. Osazuwa‐Peters , U. C. Megwalu , and N. Osazuwa‐Peters , “Association of Human Papillomavirus Status With Suicide Risk Among Patients With Head and Neck Cancer,” JAMA Otolaryngology–Head & Neck Surgery 149, no. 4 (April 2023): 291–299, 10.1001/jamaoto.2022.4839. 36795392 PMC9936382

[34] J. W. Choi , E. C. Park , T. H. Kim , and E. Han , “Mental Disorders and Suicide Risk Among Cancer Patients: A Nationwide Cohort Study,” Archives of Suicide Research 26, no. 1 (January 2022): 44–55, 10.1080/13811118.2020.1779156. 32538322

[35] D. C. Chang , A. W. Chen , Y. S. Lo , Y. C. Chuang , and M. K. Chen , “Factors Associated With Suicidal Ideation Risk in Head and Neck Cancer: A Longitudinal Study,” Laryngoscope 129, no. 11 (November 2019): 2491–2495, 10.1002/lary.27843. 30690748

[36] T. G. Chang , P. C. Huang , C. Y. Hsu , and T. T. Yen , “Demoralization in Oral Cancer Inpatients and its Association With Spiritual Needs, Quality of Life, and Suicidal Ideation: A Cross‐Sectional Study,” Health and Quality of Life Outcomes 20, no. 1 (April 2022): 60, 10.1186/s12955-022-01962-6. 35366908 PMC8976948

[37] M. Henry , J. G. Albert , S. Frenkiel , et al. “Body Image Concerns in Patients With Head and Neck Cancer: A Longitudinal Study,” Frontiers in Psychology 13 (March 2022): 816587, 10.3389/fpsyg.2022.816587. 35401366 PMC8988682

[38] M. Henry , E. Arnovitz , S. Frenkiel , et al. “Psychosocial Outcomes of Human Papillomavirus (HPV)‐and Non‐HPV‐Related Head and Neck Cancers: A Longitudinal Study,” Psycho‐Oncology 31, no. 2 (February 2022): 185–197, 10.1002/pon.5803. 35122670

[39] K. E. Henson , R. Brock , J. Charnock , B. Wickramasinghe , O. Will , and A. Pitman , “Risk of Suicide After Cancer Diagnosis in England,” JAMA Psychiatry 76, no. 1 (January 2019): 51–60, 10.1001/jamapsychiatry.2018.3181. 30476945 PMC6583458

[40] A. Heyda , A. Bieleń , A. Wygoda , and K. Składowski , “Walking Through the Valley of the Shadow of Death—The Psychotherapy of the Head and Neck Cancer Patient Expressing Suicidal Ideations and Impulses,” Journal of Clinical Psychology 79, no. 6 (June 2023): 1562–1571, 10.1002/jclp.23517. 37006196

[41] X. Hu , J. Ma , A. Jemal , et al. “Suicide Risk Among Individuals Diagnosed With Cancer in the US, 2000‐2016,” JAMA Network Open 6, no. 1 (January 2023): e2251863, 10.1001/jamanetworkopen.2022.51863. 36662522 PMC9860529

[42] F. H. Liu , J. Y. Huang , C. Lin , and T. J. Kuo , “Suicide Risk After Head and Neck Cancer Diagnosis in Taiwan: A Retrospective Cohort Study,” Journal of Affective Disorders 320 (January 2023): 610–615, 10.1016/j.jad.2022.09.151. 36198362

[43] Q. Liu , X. Wang , X. Kong , et al. “Subsequent Risk of Suicide Among 9,300,812 Cancer Survivors in US: A Population‐Based Cohort Study Covering 40 Years of Data,” eClinicalMedicine 44 (February 2022): 44, 10.1016/j.eclinm.2022.101295. PMC8850339 35198920

[44] I. M. Michalek , F. L. Caetano dos Santos , U. Wojciechowska , and J. Didkowska , “Suicide After a Diagnosis of Cancer: Follow‐Up of 1.4 Million Individuals, 2009–2019,” Cancers 15, no. 17 (August 2023): 4315, 10.3390/cancers15174315. 37686591 PMC10486959

[45] S. M. Nugent , B. J. Morasco , R. Handley , et al. “Risk of Suicidal Self‐Directed Violence Among US Veteran Survivors of Head and Neck Cancer,” JAMA Otolaryngology–Head & Neck Surgery 147, no. 11 (November 2021): 981–989, 10.1001/jamaoto.2021.2625. 34617963 PMC8498929

[46] D. Stanbouly , F. Goudarzi , R. A. Ashshi , N. Patel , S. R. Chandra , and S. K. Chuang , “Is Health Insurance a Risk Factor for Suicidal Ideation Among Adults Suffering From Head and Neck Cancer in the US?,” Oral Surgery, Oral Medicine, Oral Pathology and Oral Radiology 135, no. 4 (April 2023): 475–480, 10.1016/j.oooo.2022.08.001. 36229364

[47] M. Sun , F. Feng , J. He , S. Fan , Y. Yang , and J. Ji , “Investigation and Correlation Analysis of Financial Toxicity and Risk of Suicide in the Patients With Head and Neck Cancer,” Chinese Journal of Practical Nursing (2023): 1815–1821, 10.21203/rs.3.rs-3934310/v1.

[48] L. M. Sun , C. L. Lin , W. C. Shen , and C. H. Kao , “Suicide Attempts in Patients With Head and Neck Cancer in Taiwan,” Psycho‐Oncology 29, no. 6 (June 2020): 1026–1035, 10.1002/pon.5373. 32128937

[49] Z. Tu , C. Li , Q. Hu , and J. Luo , “Larysuicide: An Online Risk Stratification System to Identify Patients at High Risk of Suicide After the Laryngeal Cancer Diagnosis,” Journal of Cancer Research and Clinical Oncology 149, no. 9 (August 2023): 6455–6465, 10.1007/s00432-023-04635-z. 36763172 PMC11796805

[50] A. Waltho , D. Thomson , R. Pattison , J. Woolley , and T. Hawthorn , “Developing and Evaluating a Pathway for Screening and Treatment of Depression in Patients With Head and Neck Cancer,” Journal of Psychosomatic Research 141 (February 2021): 110346, 10.1016/j.jpsychores.2020.110346. 33387700

[51] N. G. Zaorsky , Y. Zhang , L. Tuanquin , S. M. Bluethmann , H. S. Park , and V. M. Chinchilli , “Suicide Among Cancer Patients,” Nature Communications 10, no. 1 (January 2019): 207, 10.1038/s41467-018-08170-1. PMC6331593 30643135

[52] E. Hem , J. H. Loge , T. Haldorsen , and Ø Ekeberg , “Suicide Risk in Cancer Patients From 1960 to 1999,” Journal of Clinical Oncology 22, no. 20 (October 2004): 4209–4216, 10.1200/jco.2004.02.052. 15483032

[53] K. Innos , K. Rahu , M. Rahu , and A. Baburin , “Suicides Among Cancer Patients in Estonia: A Population‐Based Study,” European Journal of Cancer 39, no. 15 (October 2003): 2223–2228, 10.1016/s0959-8049(03)00598-7. 14522382

[54] K. Nasseri , P. K. Mills , H. R. Mirshahidi , and L. H. Moulton , “Suicide in Cancer Patients in California, 1997–2006,” Archives of Suicide Research 16, no. 4 (October 2012): 324–333, 10.1080/13811118.2013.722056. 23137222

[55] W. Oberaigner , B. Sperner‐Unterweger , M. Fiegl , S. Geiger‐Gritsch , and C. Haring , “Increased Suicide Risk in Cancer Patients in Tyrol/Austria,” General Hospital Psychiatry 36, no. 5 (September 2014): 483–487, 10.1016/j.genhosppsych.2014.05.017. 25015541

[56] G. Smailyte , D. Jasilionis , A. Kaceniene , A. Krilaviciute , D. Ambrozaitiene , and V. Stankuniene , “Suicides Among Cancer Patients in Lithuania: A Population‐Based Census‐Linked Study,” Cancer Epidemiology 37, no. 5 (October 2013): 714–718, 10.1016/j.canep.2013.05.009. 23809215

[57] G. P. Yu , V. Mehta , D. Branovan , Q. Huang , and S. P. Schantz , “Non–Cancer‐Related Deaths From Suicide, Cardiovascular Disease, and Pneumonia in Patients With Oral Cavity and Oropharyngeal Squamous Carcinoma,” Archives of Otolaryngology—Head and Neck Surgery 138, no. 1 (January 2012): 25–32, 10.1001/archoto.2011.236. 22249625

[58] T. Akechi , H. Okamura , T. Nakano , et al. “Gender Differences in Factors Associated With Suicidal Ideation in Major Depression Among Cancer Patients,” Psycho‐Oncology 19, no. 4 (April 2010): 384–389, 10.1002/pon.1587. 19472294

[59] A. Goelitz , “Suicidal Ideation at End‐Of‐Life: The Palliative Care Team's Role,” Palliative & Supportive Care 1, no. 3 (September 2003): 275–278, 10.1017/s1478951503030244. 16594428

[60] M. Henry , Z. Rosberger , L. Bertrand , et al. “Prevalence and Risk Factors of Suicidal Ideation Among Patients With Head and Neck Cancer: Longitudinal Study,” Otolaryngology–Head and Neck Surgery 159, no. 5 (November 2018): 843–852, 10.1177/0194599818776873. 29865939

[61] A. K. Kapoor and S. Bhatnagar , “Are We Missing Out on Something?,” Indian Journal of Palliative Care 24, no. 3 (July 2018): 381–383, 10.4103/ijpc.ijpc_199_17. 30111959 PMC6069621

[62] W. S. Kendal , “Suicide and Cancer: A Gender‐Comparative Study,” Annals of Oncology 18, no. 2 (February 2007): 381–387, 10.1093/annonc/mdl385. 17053045

[63] S. Misono , N. S. Weiss , J. R. Fann , M. Redman , and B. Yueh , “Incidence of Suicide in Persons With Cancer,” Journal of Clinical Oncology 26, no. 29 (October 2008): 4731–4738, 10.1200/jco.2007.13.8941. 18695257 PMC2653137

[64] A. T. Beck and R. A. Steer , Manual for the Beck Scale for Suicide Ideation, Vol. 63 (Psychological Corporation), 1991.

[65] M. Barkham , B. Bewick , T. Mullin , et al. “The CORE‐10: A Short Measure of Psychological Distress for Routine Use in the Psychological Therapies,” Counselling and Psychotherapy Research 13, no. 1 (March 2013): 3–13, 10.1080/14733145.2012.729069.

[66] S. Zhou , J. Zhang , Y. Ye , Q. Zhang , and J. Fu , “Development of Suicide Risk Assessment Scale of Cancer Patients,” Chinese General Practice 22, no. 9 (March 2019): 1062, 10.12114/j.issn.1007-9572.2018.00.229.

[67] World Health Organisation . “Suicide Worldwide in 2019,”. [accessed: 03 April 2025], https://iris.who.int/bitstream/handle/10665/341728/9789240026643‐eng.pdf.

[68] J. O. Park , I. C. Nam , C. S. Kim , et al. “Sex Differences in the Prevalence of Head and Neck Cancers: A 10‐Year Follow‐Up Study of 10 Million Healthy People,” Cancers 14, no. 10 (May 2022): 2521, 10.3390/cancers14102521. 35626129 PMC9139445

[69] S. Bennett , K. A. Robb , R. Adán‐González , T. C. Zortea , and R. C. O’Connor , “Psychosocial Factors Distinguishing Men Who Have Attempted Suicide From Men With Suicidal Ideation and Non‐Suicidal Men: Findings From a Global Survey,” Journal of Men’s Studies 33, no. 1 (March 2025): 30–61, 10.1177/10608265241256258.

[70] A. Chandler , “Masculinities and Suicide: Unsettling ‘Talk’as a Response to Suicide in Men,” Critical Public Health 32, no. 4 (August 2022): 499–508, 10.1080/09581596.2021.1908959.

[71] D. I. Kwon and B. A. Miles , and Education Committee of the American Head and Neck Society (AHNS) , “Hypopharyngeal Carcinoma: Do You Know Your Guidelines?,” Head & Neck 41, no. 3 (March 2019): 569–576, 10.1002/hed.24752. 30570183

[72] H. E. Eckel and P. J. Bradley , “Treatment Options for Hypopharyngeal Cancer,” Hypopharyngeal Cancer 83 (2019): 47–53, 10.1159/000492308. 30943512

[73] A. Bozec , G. Poissonnet , O. Dassonville , and D. Culié , “Current Therapeutic Strategies for Patients With Hypopharyngeal Carcinoma: Oncologic and Functional Outcomes,” Journal of Clinical Medicine 12, no. 3 (February 2023): 1237, 10.3390/jcm12031237. 36769885 PMC9918098

[74] X. Luo , X. Huang , J. Luo , et al. “Induction TPF Followed by Concurrent Chemoradiotherapy Versus Concurrent Chemoradiotherapy Alone in Locally Advanced Hypopharyngeal Cancer: A Preliminary Analysis of a Randomized Phase 2 Trial,” BMC Cancer 22, no. 1 (November 2022): 1235, 10.1186/s12885-022-10306-y. 36447152 PMC9706919

[75] J. Briscoe and J. A. Webb , “Scratching the Surface of Suicide in Head and Neck Cancer,” JAMA Otolaryngology–Head & Neck Surgery 142, no. 6 (June 2016): 610, 10.1001/jamaoto.2016.0255. 27031661

[76] G. Huh , S. H. Ahn , J. G. Suk , et al. “Severe Late Dysphagia After Multimodal Treatment of Stage III/IV Laryngeal and Hypopharyngeal Cancer,” Japanese Journal of Clinical Oncology 50, no. 2 (February 2020): 185–192, 10.1093/jjco/hyz158. 31711185

[77] S. Mahalingam and P. Spielmann , “Quality of Life Outcomes Following Treatment of Hypopharyngeal Cancer,” Hypopharyngeal Cancer 83 (2019): 126–134, 10.1159/000492356. 30943471

[78] L. McDowell , K. Gough , I. White , J. Corry , and D. Rischin , “Sexual Health, Sexuality and Sexual Intimacy in Patients With Head and Neck Cancer–A Narrative Review,” Oral Oncology 157 (October 2024) 106975, 10.1016/j.oraloncology.2024.106975. 39083855

[79] S. Stack , “Contributing Factors to Suicide: Political, Social, Cultural and Economic,” Preventive Medicine 152 (November 2021): 106498, 10.1016/j.ypmed.2021.106498. 34538366

[80] L. Sharp , L. J. Watson , L. Lu , et al. “Cancer‐Related Fatigue in Head and Neck Cancer Survivors: Longitudinal Findings From the Head and Neck 5000 Prospective Clinical Cohort,” Cancers 15, no. 19 (October 2023): 4864, 10.3390/cancers15194864. 37835558 PMC10571913

[81] M. Berg , E. Silander , M. Bove , L. Johansson , J. Nyman , and E. Hammerlid , “Fatigue in Long‐Term Head and Neck Cancer Survivors From Diagnosis Until Five Years After Treatment,” Laryngoscope 133, no. 9 (September 2023): 2211–2221, 10.1002/lary.30534. 36695154

[82] S. N. Rogers , E. S. Hogg , W. K. Cheung , et al. “What Will I Be Like’After My Diagnosis of Head and Neck Cancer?,” European Archives of Oto‐Rhino‐Laryngology 272, no. 9 (September 2015): 2463–2472, 10.1007/s00405-014-3189-x. 25047397

[83] R. Andreassen , B. Jönsson , and E. Hadler‐Olsen , “Oral Health Related Quality of Life in Long‐Term Survivors of Head and Neck Cancer Compared to a General Population From the Seventh Tromsø Study,” BMC Oral Health 22, no. 1 (March 2022): 100, 10.1186/s12903-022-02140-2. 35354441 PMC8969380

[84] S. M. Strayhorn , L. R. Carnahan , K. Zimmermann , et al. “Comorbidities, Treatment‐Related Consequences, and Health‐Related Quality of Life Among Rural Cancer Survivors,” Supportive Care in Cancer 28, no. 4 (April 2020): 1839–1848, 10.1007/s00520-019-05005-7. 31342166 PMC6980904

[85] A. Molassiotis and M. Rogers , “Symptom Experience and Regaining Normality in the First Year Following a Diagnosis of Head and Neck Cancer: A Qualitative Longitudinal Study,” Palliative & Supportive Care 10, no. 3 (September 2012): 197–204, 10.1017/s147895151200020x. 22613011

[86] C. J. Semple , L. Dunwoody , G. W. Kernohan , E. McCaughan , and K. Sullivan , “Changes and Challenges to Patients’ Lifestyle Patterns Following Treatment for Head and Neck Cancer,” Journal of Advanced Nursing 63, no. 1 (July 2008): 85–93, 10.1111/j.1365-2648.2008.04698.x. 18598253

[87] A. N. Aminnudin , J. G. Doss , S. M. Ismail , et al. “Can Post‐Treatment Oral Cancer Patients’ Concerns Reflect Their Cancer Characteristics, HRQoL, Psychological Distress Level and Satisfaction With Consultation?,” ecancermedicalscience 14 (2020): 1118, 10.3332/ecancer.2020.1118. 33209109 PMC7652548

[88] G. K. Halkett , R. M. Golding , D. Langbecker , et al. “From the Carer's Mouth: A Phenomenological Exploration of Carer Experiences With Head and Neck Cancer Patients,” Psycho‐Oncology 29, no. 10 (October 2020): 1695–1703, 10.1002/pon.5511. 32779257

[89] R. S. Parahoo , C. J. Semple , S. Killough , and E. McCaughan , “The Experience Among Patients With Multiple Dental Loss as a Consequence of Treatment for Head and Neck Cancer: A Qualitative Study,” Journal of Dentistry 82 (March 2019): 30–37, 10.1016/j.jdent.2019.01.010. 30710651

[90] J. Riggauer , D. Blaser , O. Elicin , B. Gahl , R. Giger , and S. A. Mueller , “Risk Factors for Fear of Recurrence in Head and Neck Cancer Patients,” Laryngoscope 133, no. 7 (July 2023): 1630–1637, 10.1002/lary.30340. 36054694

[91] S. L. Tsay , J. Y. Wang , Y. H. Lee , and Y. J. Chen , “Fear of Recurrence: A Mediator of the Relationship Between Physical Symptoms and Quality of Life in Head and Neck Cancer Patients,” European Journal of Cancer Care 29, no. 4 (July 2020): e13243, 10.1111/ecc.13243. 32510671

[92] S. N. Rogers , B. Scott , D. Lowe , G. Ozakinci , and G. M. Humphris , “Fear of Recurrence Following Head and Neck Cancer in the Outpatient Clinic,” European Archives of Oto‐Rhino‐Laryngology 267, no. 12 (December 2010): 1943–1949, 10.1007/s00405-010-1307-y. 20582704

[93] B. H. Campbell , A. Marbella , and P. M. Layde , “Candidate's Thesis: Quality of Life and Recurrence Concern in Survivors of Head and Neck Cancer,” Laryngoscope 110, no. 6 (June 2000): 895–906, 10.1097/00005537-200006000-00003. 10852502

[94] S. Aghajanzadeh , T. Karlsson , L. Tuomi , M. Engström , and C. Finizia , “Trismus, Health‐Related Quality of Life, and Trismus‐Related Symptoms up to 5 Years Post‐Radiotherapy for Head and Neck Cancer Treated Between 2007 and 2012,” Supportive Care in Cancer 31, no. 3 (March 2023): 166, 10.1007/s00520-023-07605-w. 36781552 PMC9925520

[95] S. N. Rogers , A. E. Waylen , S. Thomas , et al. “Quality of Life, Cognitive, Physical and Emotional Function at Diagnosis Predicts Head and Neck Cancer Survival: Analysis of Cases From the Head and Neck 5000 Study,” European Archives of Oto‐Rhino‐Laryngology 277, no. 5 (May 2020): 1515–1523, 10.1007/s00405-020-05850-x. 32062743 PMC7160091

[96] C. J. Semple , G. McKenna , R. Parahoo , S. N. Rogers , and Y. T. Ehrsson , “Factors That Affect Quality of Life for Older People With Head and Neck Cancer: A Systematic Review,” European Journal of Oncology Nursing 63 (April 2023): 102280, 10.1016/j.ejon.2023.102280. 36893570

[97] T. Hafford‐Letchfield , H. Gleeson , P. Ryan , et al. “‘He Just Gave up’: An Exploratory Study Into the Perspectives of Paid Carers on Supporting Older People Living in Care Homes With Depression, Self‐Harm, and Suicide Ideation and Behaviours,” Ageing and Society 40, no. 5 (May 2020): 984–1003, 10.1017/S0144686X18001447.

[98] K. Kvåle and O. Synnes , “Living With Life‐Prolonging Chemotherapy—Control and Meaning‐Making in the Tension Between Life and Death,” European Journal of Cancer Care 27, no. 1 (January 2018): e12770, 10.1111/ecc.12770. 28892215

[99] S. Silva , A. Bartolo , I. M. Santos , A. Pereira , and S. Monteiro , “Towards a Better Understanding of the Factors Associated With Distress in Elderly Cancer Patients: A Systematic Review,” International Journal of Environmental Research and Public Health 19, no. 6 (March 2022): 3424, 10.3390/ijerph19063424. 35329112 PMC8949443

[100] P. Saini , J. McIntyre , R. Corcoran , et al. “Predictors of Emergency Department and GP Use Among Patients With Mental Health Conditions: A Public Health Survey,” British Journal of General Practice 70, no. 690 (January 2020): e1–e8, 10.3399/bjgp19X707093. PMC6917360 31848197

[101] E. A. Boakye , K. J. Sykes , J. L. Hamilton , et al. “Head and Neck Oncology Professionals’ Perceptions of Suicide Risk Screening Among Patients,” Oral Oncology 151 (April 2024): 106728, 10.1016/j.oraloncology.2024.106728. 38402846

[102] B. Kansara , A. Basta , M. Mikhael , et al. “Suicide Risk Screening for Head and Neck Cancer Patients: An Implementation Study,” Applied Clinical Informatics 15, no. 2 (March 2024): 404–413, 10.1055/s-0044-1787006. 38777326 PMC11111312

[103] L. Granek , O. Nakash , M. Ben‐David , S. Shapira , and S. Ariad , “Oncologists', Nurses', and Social Workers' Strategies and Barriers to Identifying Suicide Risk in Cancer Patients,” Psycho‐Oncology 27, no. 1 (January 2018): 148–154, 10.1002/pon.4481. 28635073

[104] J. M. Anderson , R. Gibbison , J. A. Twigg , and A. Kanatas , “Development of a Protocol for Assessment of Suicide Risk in Patients With Head and Neck Cancer,” British Journal of Oral and Maxillofacial Surgery 59, no. 1 (January 2021): e23–e26, 10.1016/j.bjoms.2020.08.004. 33131803

[105] C. Lin , S. Y. Kang , S. Donermeyer , T. N. Teknos , and S. M. Wells‐Di Gregorio , “Supportive Care Needs of Patients With Head and Neck Cancer Referred to Palliative Medicine,” Otolaryngology–Head and Neck Surgery 163, no. 2 (August 2020): 356–363, 10.1177/0194599820912029. 32178571

[106] E. Saghafi , C. A. Andås , J. Bernson , and G. Kjeller , “Patients’ Experiences of Adverse Symptoms, Emotions, and Coping Strategies in Connection to Treatment of Head and Neck Cancer‐An Interview Study,” BMC Oral Health 23, no. 1 (September 2023): 641, 10.1186/s12903-023-03366-4. 37670339 PMC10478420

[107] A. E. Richardson , E. Broadbent , and R. P. Morton , “A Systematic Review of Psychological Interventions for Patients With Head and Neck Cancer,” Supportive Care in Cancer 27, no. 6 (June 2019): 2007–2021, 10.1007/s00520-019-04768-3. 30937599

[108] E. Semenenko , S. Banerjee , I. Olver , and P. Ashinze , “Review of Psychological Interventions in Patients With Cancer,” Supportive Care in Cancer 31, no. 4 (April 2023): 210, 10.1007/s00520-023-07675-w. 36913136

[109] S. N. Rogers and D. Lowe , “An Evaluation of the Head and Neck Cancer Patient Concerns Inventory Across the Merseyside and Cheshire Network,” British Journal of Oral and Maxillofacial Surgery 52, no. 7 (September 2014): 615–623, 10.1016/j.bjoms.2014.04.011. 24927654

[110] S. N. Rogers , C. Allmark , F. Bekiroglu , et al. “Improving Quality of Life Through the Routine Use of the Patient Concerns Inventory for Head and Neck Cancer Patients: Main Results of a Cluster Preference Randomised Controlled Trial,” European Archives of Oto‐Rhino‐Laryngology 278, no. 9 (September 2021): 3435–3449, 10.1007/s00405-022-07499-0. 33346856 PMC7751263

[111] C. J. Semple , D. Lannon , E. Qudairat , E. McCaughan , and R. McCormac , “Development and Evaluation of a Holistic Surgical Head and Neck Cancer Post‐Treatment Follow‐Up Clinic Using Touchscreen Technology—Feasibility Study,” European Journal of Cancer Care 27, no. 2 (March 2018): e12809, 10.1111/ecc.12809. 29419940

[112] S. Peters , A. Rogers , P. Salmon , et al. “What Do Patients Choose to Tell Their Doctors? Qualitative Analysis of Potential Barriers to Reattributing Medically Unexplained Symptoms,” Journal of General Internal Medicine 24, no. 4 (April 2009): 443–449, 10.1007/s11606-008-0872-x. 19089505 PMC2659146

[113] A. Barsouk , J. S. Aluru , P. Rawla , K. Saginala , and A. Barsouk , “Epidemiology, Risk Factors, and Prevention of Head and Neck Squamous Cell Carcinoma,” Medical Sciences 11, no. 2 (June 2023): 42, 10.3390/medsci11020042. 37367741 PMC10304137

[114] National Institute for Health and Care Excellence . “Quality Statement 5: Supporting People Bereaved or Affected by a Suspected Suicide,” 2019. Accessed: 16 October 2024, https://www.nice.org.uk/guidance/qs189/chapter/Quality‐statement‐5‐Supporting‐people‐bereaved‐or‐affected‐by‐a‐suspected‐suicide.

[115] World Health Organization . “Suicide Worldwide in 2019: Global Health Estimates,” 2021. Accessed: 16 October 2024, https://iris.who.int/bitstream/handle/10665/341728/9789240026643‐eng.pdf?sequence=1.

[116] Organisation for Economic Co‐operation and Development . “ODA Recipiences: Countries, Territories, and International Organisation,”. Accessed, October 16, 2024, https://www.oecd.org/en/topics/sub‐issues/oda‐eligibility‐and‐conditions/dac‐list‐of‐oda‐recipients.html.

[117] S. N. Rogers , D. Lowe , and A. Kanatas , “Social Determinants of Health‐Related Quality of Life Outcomes for Head and Neck Cancer Patients,” Oral 1, no. 4 (November 2021): 313–325, 10.3390/oral1040031.

[118] J. A. Twigg , J. M. Anderson , G. Humphris , I. Nixon , S. N. Rogers , and A. Kanatas , “Best Practice in Reducing the Suicide Risk in Head and Neck Cancer Patients: A Structured Review,” British Journal of Oral and Maxillofacial Surgery 58, no. 9 (November 2020): e6–e15, 10.1016/j.bjoms.2020.06.035. 32682651

[119] D. G. Deschler , J. D. Richmon , S. S. Khariwala , R. L. Ferris , and M. B. Wang , “The ‘New’ Head and Neck Cancer Patient—Young, Nonsmoker, Nondrinker, and HPV Positive: Evaluation,” Otolaryngology‐Head and Neck Surgery 151, no. 3 (September 2014): 375–380, 10.1177/0194599814538605. 24925311 PMC4894657

[120] S. N. Rogers , H. H. Tsai , M. G. Cherry , J. M. Patterson , and C. J. Semple , “Experiences and Needs of Carers of Patients With Head and Neck Cancer: A Systematic Review,” Psycho‐Oncology 33, no. 10 (October 2024): e9308, 10.1002/pon.9308. 39334532

[121] N. Darvishi , J. Poorolajal , B. Azmi‐Naei , and M. Farhadi , “The Role of Social Support in Preventing Suicidal Ideations and Behaviors: A Systematic Review and Meta‐Analysis,” Journal of Research in Health Sciences 24, no. 2 (2024): e00609, 10.34172/jrhs.2024.144. 39072545 PMC11264453

